# Genetic identification of Árpád Dynasty members from the ossuary of the Royal Basilica at Székesfehérvár

**DOI:** 10.1016/j.isci.2026.116365

**Published:** 2026-06-12

**Authors:** Bence Kovács, Judit Olasz, Zoltán Maróti, Oszkár Schütz, Nicholas Rouse, Michael F. Nagy, Alexandra Gînguță, Kitti Maár, Balázs Tihanyi, Luca Kis, Balázs Holczmann, Balázs Kertész, Zoltán Szabó, Zsolt Bernert, Endre Neparáczki, Tibor Török, Miklós Kásler, Péter L. Nagy, Gergely I.B. Varga

**Affiliations:** 1 Department of Archaeogenetics, Institute of Hungarian Research, Budapest, Hungary; 2 Department of Genetics, University of Szeged, Szeged, Hungary; 3 Department of Pediatrics and Pediatric Health Center, University of Szeged, Szeged, Hungary; 4 Praxis Genomics LLC, Atlanta, GA, USA; 5 Hungarian Natural History Museum, Molecular Taxonomy Laboratory, Budapest, Hungary; 6 Department of Biological Anthropology, University of Szeged, Szeged, Hungary; 7 SAP Hungary Kft., Budapest, Hungary; 8 Department of History, Institute of Hungarian Research, Budapest, Hungary; 9 Szakra Stúdió Kft., Budakeszi, Hungary; 10 Hungarian Natural History Museum, Budapest, Hungary

**Keywords:** Genetics, Human genetics, Archeology, History

## Abstract

Shotgun sequencing of >400 genomes from the ossuary of the Royal Basilica of Székesfehérvár identified three additional skeletal remains carrying the Árpád Dynasty’s Y chromosome haplogroup R-ARP, raising the total known R-ARP+ individuals from four to seven. Kinship and IBD analyses placed these individuals within the dynasty alongside Béla, Duke of Macsó (†1272)—a Rurikid prince and great-great-grandson of King Béla III— and a fetus from a tomb adjacent to Béla III. One of these is King Béla II “the Blind” (1131–1141); a second is a likely second-degree relative of St. Ladislaus (probably an uncle, identity unresolved); the third, buried outside the original Basilica walls, is related to the Árpáds only by Y-haplogroup. The authenticity of Béla, Duke of Macsó’s remains was genetically confirmed. IBD also linked Árpáds to conquering Hungarians, Vikings, and the Aba, Báthory, and Corvinus families.

## Highlights

• Three additional R-ARP+ Árpád lineage carriers identified from 400+ Royal Basilica genomes
• King Béla II “the Blindˮ reliably identified by autosomal IBD
• Authenticity of Béla, Duke of Macsó’s remains genetically confirmed
• IBD ties Árpáds to conquering Hungarians, Vikings, Abas, Báthorys, Corvinus

## Graphical abstract

**Figure:**
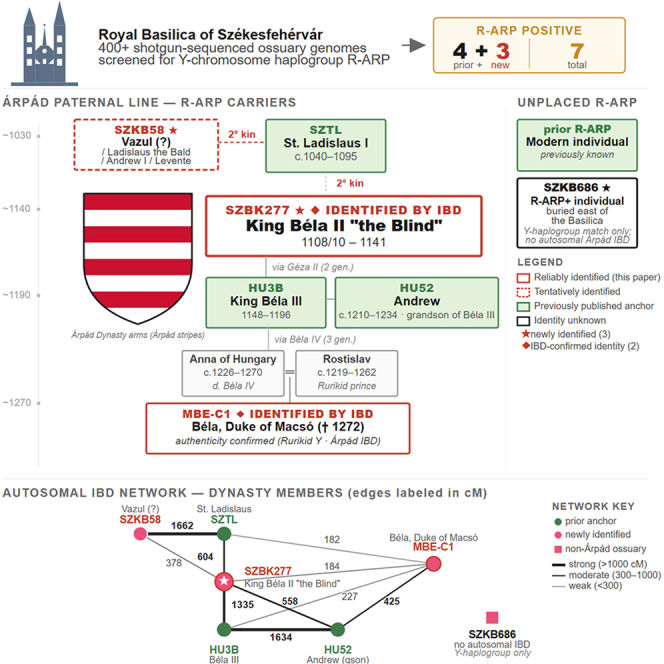

## Introduction

The first historically verifiable member of the ruling dynasty known as the Árpád Dynasty was Álmos (d. 895), who prior to 895 served as one of the leaders of the Hungarian tribal confederation. The dynasty, however, was not named after him but after his son, Prince Árpád (d. ca. 900); a designation that emerged in Hungarian historiography during the 18th century. It was under Árpád’s leadership that the Hungarians migrated into the Carpathian Basin at the end of the 9th century. The foundations of the Christian Kingdom of Hungary were laid primarily by his great-great grandson, Stephen I, the dynasty’s first king (later canonized as Saint Stephen, reigned 1000/1001–1038). The Árpád dynasty, which played a formative role in the history of Central Europe, ended with King Andrew III (1290–1301) in the year 1301.^1^^,^^2^

**Figure 1:**
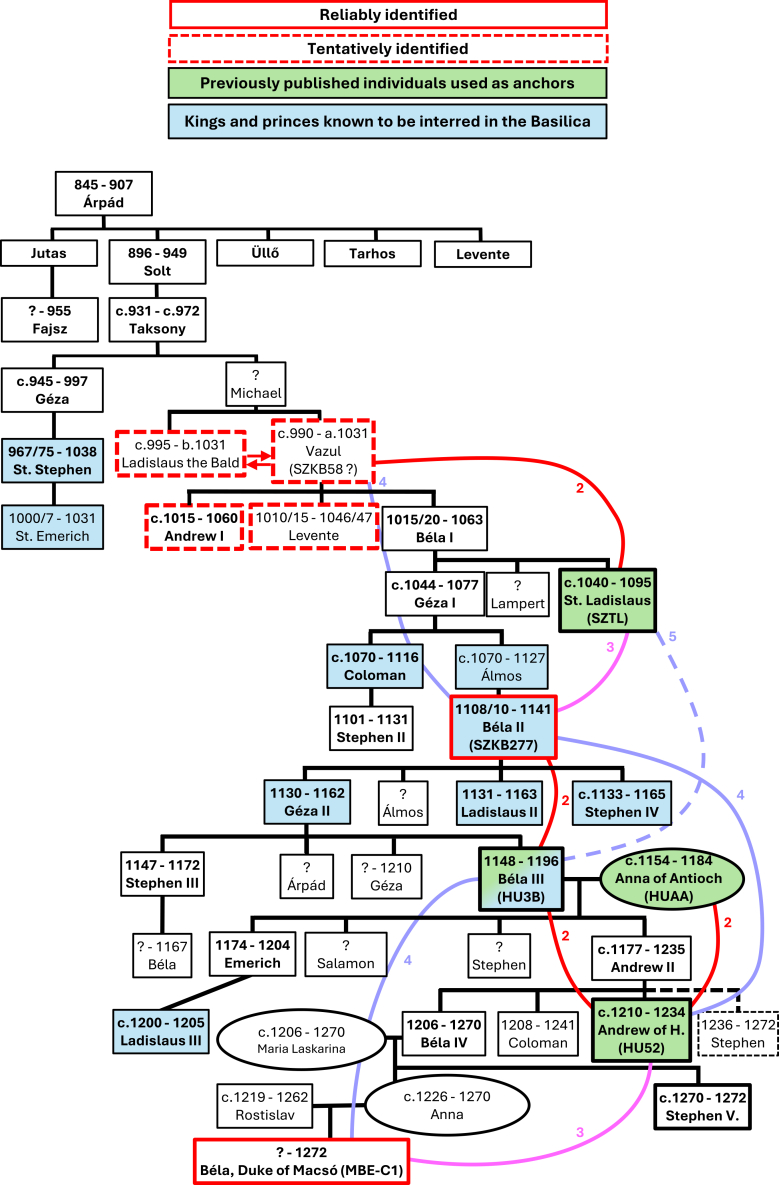
Abridged version of the Árpád Dynasty family tree Blue squares denote kings and princes known to be interred in the basilica. Top numbers indicate dates of birth and death. Green squares mark previously published individuals. Solid red outlines indicate individuals identified in this current paper. Dashed red outlines indicate individuals tentatively identified. Results of kinship analysis are indicated with solid lines connecting the squares (2nd degree—red; 3rd degree—pink; 4th degree—purple). The dashed purple line indicates likely 5th degree relationship.

Stephen I (Saint Stephen) was buried in one of his seats of power, Székesfehérvár, in the Church of the Virgin Mary that he founded. After Saint Stephen’s death, this church, commonly known as the Royal Basilica of Székesfehérvár, served as the coronation and burial site for Hungarian kings until the Ottoman occupation of the city in 1543.^3^ Out of the 37 Hungarian monarchs who died before this event, 15 are known to be interred in the basilica. Historical sources indicate that at least ten Árpáds, eight kings, and two princes, were laid to rest here^4^ (Figure 1).

**Figure 2:**
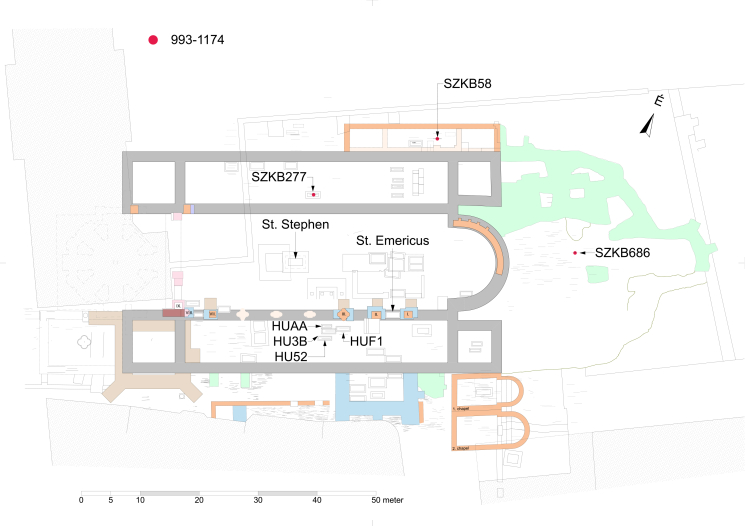
Layout of the reconstructed foundation of the Royal Basilica of Székesfehérvár The gray mottled areas represent current buildings overlaying the site. The dark gray shade indicates the earliest construction, dating back to the 11th century. Orange indicates further additions from the 12th century. Blue indicates the Anjou Chapel from the 14th century. Green indicates the addition by Matthias Corvin from the 15th century. The position of the individuals buried here is indicated with thin lines. The R-ARP positive individuals are indicated with red dots. All remains discussed in the work are described with their working abbreviations (HUAA: Anna of Antioch, HU3B: Béla III, HU52: possibly Prince Andrew, SZKB277: Béla II (The Blind), SZKB58: second degree relative of Saint Ladislaus, SZKB686: unknown male, HUF-1: unknown fetus).

During the following centuries of war and neglect, the basilica fell into ruin, and the only royal graves left undisturbed were those of King Béla III (1172–1196) and his first spouse, Queen Anna of Antioch (†1184/85) (Figures 2; S1). Their tombs and three other tombs in their immediate vicinity were discovered by accident during the digging of a drainage canal in 1848.^5^^,^^6^ One of the adjacent tombs contained a pregnant female. The royal couple, one of the adjacent male skeletons (HU52), and the fetus of the pregnant woman were later reinterred in the Matthias Church of Buda.^7^ Genetic analysis of the remains of King Béla III and Queen Anna of Antioch as well as HU52 were previously analyzed and published,^8^^,^^9^ while analysis of the remains of the fetus (HUF-1) are included in the present study (Figures 2 and S1).

Shotgun sequencing of the remains of King Béla III and HU52 from the Matthias Church identified their Y chromosome haplogroup as a derivative of the R1a haplogroup (R1a-M420>R-M459>R-M198>R-M417>R-PF6162>R-Z93>R-Z94>R-Z2124>R-Z2125>R-Z2123>R-Y20746>R-Y2632>R-Y2633>R-SUR51).^8^ Analysis of a living relative (UVD) allowed identification of private SNPs found in King Béla III and HU52 with confidence. Nine private SNPs were described to define the R-ARP haplogroup.^8^ Similar studies of the relic of St. Ladislaus held in the Héderváry Chapel of the Basilica of Győr, confirmed that the skull in the herma of St. Ladislaus shared the same Y chromosome haplogroup.^10^ This paper by Varga et al. also identified HU52 as the grandson of Béla III and Anna of Antioch, most likely Prince Andrew of Halych, son of Andrew II, who lived c.1210–1234. Based on these results, we undertook a search for additional royal family members among the other skeletal remains of the Ossuary of Székesfehérvár Royal Basilica to help us better delineate the family tree of the Árpád Dynasty. The remains of approximately 935 individuals unearthed inside and around the basilica in excavations conducted between 1936 and 2002 were collected at the National Memorial-Medieval Ruin Garden in Székesfehérvár.^11^ Our research team analyzed samples belonging to more than 600 individuals that had an associated skull, of which more than 400 underwent whole-genome sequencing, resulting in the identification of three additional R-ARP-positive remains, SZKB58, SZKB277, and SZKB686.^11^ The current work will focus on the characterization of the R-ARP positive individuals and their known or suspected relatives, while population genetics analysis of the entire dataset will be published elsewhere. We used kinship and IBD analysis to establish the familial relationships of the R-ARP positive remains and the previously identified Árpád Dynasty members: Ladislaus I, Béla III, Prince Andrew of Halych, son of Andrew II (HU52) and Béla, Duke of Macsó and Bosnia.^12^ The latter was the grandson of King Béla IV, son of Princess Anna (daughter of King Béla IV), and Rostislav, Lord of Macsó (1247/1254–1262) a member of the Rurikid Dynasty (Figure 1). He was murdered by oligarchs in 1272. His skeleton was discovered in 1915 during the excavation of the sacristy at the Margaret Island Monastery^13^ and is currently housed in the Hungarian Natural History Museum. His remains were included in the relatedness analysis since he was reliably identified through archaeological, and anthropological characteristics and we wanted to provide genetic confirmation for his identity as well.^12^^,^^14^^,^^15^ The unidentified fetal remains from the tomb adjacent to Béla III were also included in the analysis.

## Results and discussion

### Identification of three additional R-ARP individuals

We discovered three human remains, SZKB58, SZKB277, and SZKB686 belonging to the R-ARP haplogroup (R1a1a1b2a2a1c3a∼), previously described in Árpád Dynasty individuals Béla III (HU3B), HU52, St. Ladislaus (SZTL) (Table S1), and a present-day individual from Újvidék (Novi Sad, Serbia) (UVD).^8^^,^^9^^,^^10^ Their nomenclature and key characteristics are described in Table S2. Their photographs and the results of their anthropological examinations based on the description provided by Érdy^5^ and Éry et al.^11^ are given in the STAR Methods section. The NGS statistics of the representatives of the Árpád paternal lineage, together with the statistics of the previously published reanalyzed family members, are shown in Table S3. The haplogroup determination by Yleaf could be further derived using manual assessment of the data based on Family Tree haplogroup and SNP information (Table 1). The mitochondrial haplogroup analysis results are shown in Table S4. The originally described nine SNPs defining the R-ARP haplogroup described in Béla III and HU52 (Andrew of Halych?) were ARP2, ARP3, ARP5, ARP6, ARP8, ARP9, ARP1, ARP4, ARP7.^8^ In our current analysis, we identified two additional SNPs, ARP11 and ARP10. Based on the SNPs found in the seven known R-ARP individuals, the R-ARP haplogroup can be split into sub-haplogroups R-ARP5, R-ARP1, R-ARP10, and R-ARP11, thus providing information about the sequential appearance of all 11 R-ARP SNPs (Table 1) (Figure S2).

**Table 1:** Analysis of the R-ARP positive remains for the 11 ARP SNPs splits the R-ARP haplogroup into haplogroups: R-ARP5, R-ARP1, R-ARP11, and R-ARP10

Hg	SNP	Position_hg19	Position_hg38	Ancestr. (A)	Deriv. (D)	SZKB686	UVD	SZKB58	SZTL	SZKB277	HU3B	HU52
R-ARP5	ARP5	18224864	16112984	G	C	D	D	D	D	D	D	D
R-ARP5	ARP2	16029270	13917390	T	C	D	D	D	D	D	D	D?
R-ARP5	ARP3	17396294	15284414	T	DelT	D	D	D	D	D	D	D?
R-ARP5	ARP6	19228895	17117015	C	A	D	D	D	D	D	D?	D
R-ARP5	ARP8	21952638	19790752	T	A	D	D	D	D	D	D?	D
R-ARP5	ARP9	22471532	20309646	A	C	D	D	D?	D	D	D	D
R-ARP1	ARP1	7984982	8116941	C	T	A	D	D	D	D	D	D
R-ARP1	ARP4	18109049	15997169	C	T	A	D	D	D	D	D	D?
R-ARP1	ARP7	19318466	17206586	G	A	A	D	D	D	D	D	D
R-ARP11	ARP11	16283314	14171434	T	C	A	A	D	D	D	D	D
R-ARP10	ARP10	8693597	8825556	A	DelA	A	A	A	A	A	D	D

(A) Ancestral; (D) Derived; (D?) Not covered but deduced to be derived.

### Confirmation of the Rurikid origin of Béla, Duke of Macsó

Y chromosome analysis of Béla, Duke of Macsó showed haplogroup N1a1a1a1a1a1a7a∼ (N-Y4338). This was identical to the described haplogroup of Prince Dmitry Alexandrovich (died 1294), a seventh-degree descendant of the prominent Kievan Rus ruler Yaroslav the Wise, from the Rurik Dynasty. He was himself a fourth-degree descendant of Rurik, the founder of the dynasty. Thus, we confirm that Prince Dmitry Alexandrovich and Béla, Duke of Macsó shared a common paternal ancestry, in agreement with historical records and prior genetic analysis of Prince Dmitry Alexandrovich^15^^,^^16^ (Figures 1 and S3). Béla, Duke of Macsó is the second Rurikid prince characterized to date. The Y chromosome data confirms the previous anthropological and archaeological identification of Béla, Duke of Macsó’s remains^12^^,^^14^ and the previous determination of the Y chromosome haplogroup of the Rurik dynasty as N1a1a1a1a1a1a7a∼.

### Personal identification of Árpád Dynasty members

To position the Árpádian Y chromosome samples within the known family tree, we conducted relatedness analyses using the correctKin method (Table S5) and IBD analysis (Table S6). CorrectKin is capable of determination of close relatedness up to 4–5^th^ degree from even as low genome coverage as 0.1*x*. IBD detects direct genetic links up to 8–9^th^ generation distance or even farther. By combining the two tools, we could analyze narrow family connections and more distant kin relationships at once. Due to low average genome coverage, HUAA could not be imputed, thus she was excluded from the IBD analysis. Results are summarized in Table 2. Both analyses confirmed the previously reported kin relations among SZTL, HU3B, HUAA, and HU52^10^ (Figure 1). We note that HU52 (Andrew of Halych) shared longer total IBD with SZTL than is expected from the family tree, and longer than HU3B shares with SZTL, who is a closer relative of the saint king and shares appropriate amount of IBD with him compared to their degree of kinship. This apparent contradiction can be resolved from historical knowledge, that Gertrude of Merania, wife of king Andreas II and mother of HU52, was a descendant of an early Árpád Dynasty king Béla I as well, thus HU52 could have inherited IBDs shared with SZTL from both the maternal and paternal sides (Figure S4).

**Table 2:** Degrees of relatedness between the Árpád family members, determined with various software tools

ID1	ID2	CorrectKin coeff.	Estimated relatedness	Sum IBD >8.0 cM	Estimated relatedness (sum IBD)	Assumed degree on the family tree
HU3B	HU52	0.124528	2ND	1634.2819	2ND	2ND
HU3B	HUAA	−0.009432	uncertain	NA	NA	unrelated
HU3B	SZKB277	0.098995	2ND	1335.0353	2ND	2ND
HU3B	SZTL	0.015268	uncertain	95.5784	6th	5th
HU3B	SZKB58	0.005226	uncertain	25.4512	8th	7th
HU3B	MBE-C1	0.033192	4th	400.1541	4th	4th
HU52	HUAA	0.103061	2ND	NA	NA	2ND
HU52	SZKB277	0.045730	4th	558.1427	4th	4th
HU52	SZTL	0.011722	uncertain	184.0171	5th	7th
HU52	MBE-C1	0.038382	4th	425.3293	4th	3rd
HU52	SZKB58	−0.000511	uncertain	56.6216	7th	7th
HUAA∗	SZTL	−0.003489	uncertain	NA	NA	unrelated
HUAA∗	SZKB58	−0.001657	uncertain	NA	NA	unrelated
HUAA∗	MBE-C1	0.012413	uncertain	NA	NA	4th
HUAA∗	SZKB277	0.001457	uncertain	NA	NA	unrelated
SZTL	MBE-C1	0.013197	uncertain	182.0565	5th	9th
SZTL	SZKB58	0.133656	2ND	1661.8431	2ND	2ND
SZTL	SZKB277	0.050351	3rd	604.363	3rd	3rd
MBE-C1	SZKB58	0.001334	uncertain	25.9525	8th	11th
MBE-C1	SZKB277	0.015931	uncertain	227.3194	NA	6th
SZKB58	SZKB277	0.034163	4th	377.8829	4th	5th

Similar phenomenon was observed in the case of Béla, Duke of Macsó, who also shared longer total IBD with SZTL than expected. However, as he was also the descendant of Andreas II and Gertrude of Merania, the same hypothesis applies to his case, as well.

Both methods determined SZKB277 as second-degree relative of Béla III. and third-degree relative of St. Ladislaus, which clearly identifies the individual as Béla II (The Blind). Between SZKB277 and HU52, correctKin predicted a third-degree relationship, whereas only the IBD analysis revealed the expected fourth-degree link. This discrepancy is most likely explained by the above-mentioned multiple relatedness between these individuals (Figure S4).

Although genetic data definitely identify SZKB277 as Béla II (c. 1108–1141), radiocarbon dating yielded widely varying results for this individual, with measurements from Debrecen suggesting a death date more than a century earlier (Table 3).

**Table 3:** 14C data of the R-ARP individuals

Genetic lab ID	Type of sample	Uncalibrated BP	Error +/−	OxCal^1^ 95% cal (CE)	Measuring laboratory^2^	AMS lab ID
SZKB58	rib	980	30	995–1005 (2.8%); 1016–1158 (92.7%)	University of Georgia	UGAMS 66650
SZKB58	rib	1036	15	992–1026 (95.4%)	INR Debrecen	DeA-45540
SZKB277	petrous bone	990	25	994–1007 (6%); 1015–1051 (40.2%); 1080–1154 (49.2%)	University of Georgia	UGAMS 66649
SZKB277	petrous bone	1030	12	993–1026 (95.4%)	INR Debrecen	DeA-39139
SZKB686	tooth	930	25	1033–1174 (95.4%)	University of Georgia	UGAMS 66651
SZKB686	tooth	959	13	1031–1052 (19.2%); 1079–1154 (76.3%)	INR Debrecen	DeA-39140

Calibration was performed with OxCal 4.4, with settings IntCal20. The data are provided by two independent laboratories: INR Debrecen, AMS laboratory of the Institute for Nuclear Research, Hungarian Academy of Sciences, Debrecen, Hungary; and the University of Georgia, Radiocarbon AMS facility of the Center for Applied Isotope Studies, Georgia, United States.

A repeated analysis conducted by an independent laboratory produced more consistent results, highlighting the potential for significant deviation in radiocarbon dating outcomes.

The SZKB58 individual was identified as a second-degree relative of St. Ladislaus and a fourth-degree relative of SZKB277 (Béla II), which, based on known family relationships, indicates that the individual could be either St. Ladislaus’s grandfather (Vazul) or his uncle, King Andrew I or Prince Levente. However, some sources suggest that St. Ladislas’ grandfather was actually Ladislaus the Bald, brother of Vazul.^17^^,^^18^^,^^19^ Historical records suggest that King Andrew I was buried at Tihany, and Prince Levente was buried at Taksony.^20^

**Figure 3:**
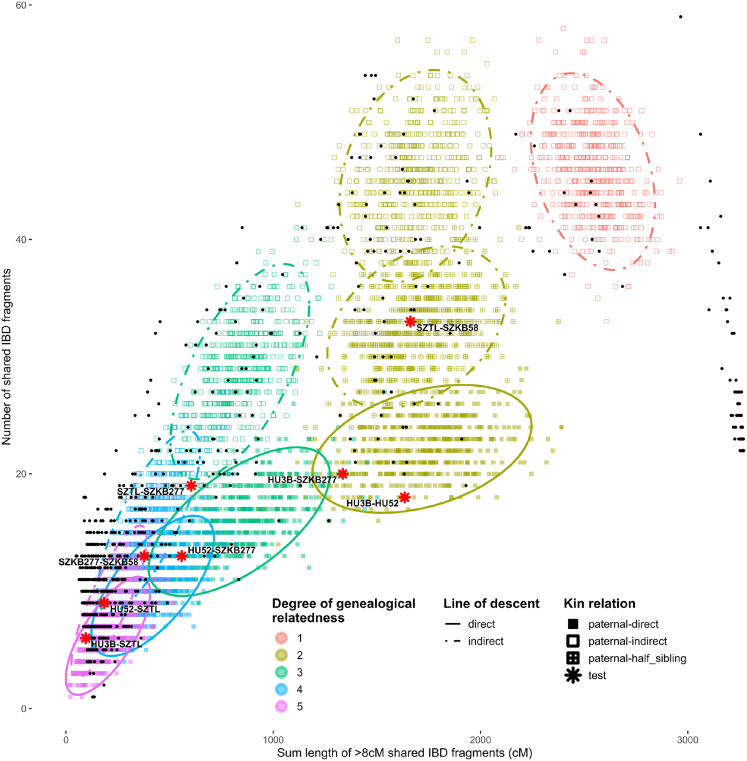
Distribution of the total shared IBD length as a function of segment number across various direct and indirect paternal familial relationships, simulated using the ibdsim2 software package from the pedsuite package collection^22^ Different generations are distinguished by colors as indicated in the figure. Direct relationships are highlighted with solid oval outlines, while indirect relationships are enclosed by dash-dotted oval outlines. Black dots represent observed >100 cM experimental connections among 2070 ancient genomes from the IBD dataset published in Schütz et al.^23^

Since kinship analysis could not unequivocally identify the SZKB58 individual, we conducted further IBD analysis. This approach was based on the principle that grandparent-grandchild (direct second-degree) relationships can, in theory, be distinguished from indirect uncle-nephew relationships by examining the number and size distribution of shared IBD segments. The distinction is possible because direct descent involves fewer meioses than indirect descent, leading to the expectation of smaller and more numerous IBD fragments in the latter case.^21^ We analyzed the total length of shared IBD segments between individuals as a function of the number of IBD segments, following the approach of Ringbauer et al. 2024.^21^ Though in that study, direct and indirect descendants did not form distinct clusters, when only paternal relationships were considered, individuals from different lines of descent separated more clearly. We conducted an extensive simulation to produce a background database of known paternal relationships, and we plotted close genealogical connections from our own database of 2070 ancient genomes on this background. Although we do not possess information on the exact genealogical distance or the line of descent in our experimental database, the distribution of the two types of data overlapped sufficiently to warrant further investigation. Finally, we plotted the known paternal kin relationships among the individuals analyzed in this study (Figure 3). Based on segment length and count parameters, the connection between Saint Ladislaus (SZTL) and SZKB58 clearly falls outside the quite discrete and characteristic distribution of direct 2nd degree paternal descent (indicated with solid line on Figure 3), suggesting an indirect connection. In contrast, the known direct paternal relations (HU3B-SZKB277: 2nd degree, HU3B-HU52: 2nd degree, HU52-SZKB277: 4th degree) aligned well with the simulated data, matching their known genealogical distances. Consequently, the likelihood that SZKB58 was St. Ladislaus’s grandparent Vazul or Ladislaus the Bald is low. A more plausible scenario is that SZKB58 was either St. Ladislaus’s paternal uncle or an undocumented half-brother. The coin found near SZKB58 (Figure S7), dating to the reign of St. Stephen (1000–1038) and Peter Orseolo (1038–1041, 1044–1046), the second king of Hungary, better fits the time frame of the uncles. However, historical sources record his uncles as King Andrew I, buried in the Benedictine Abbey of Tihany, and Levente, interred in the village of Taksony. As the genetic evidence appears to contradict the historical record, further analyses and the potential identification of additional dynasty members will be necessary to refine this relationship.

**Figure 4:**
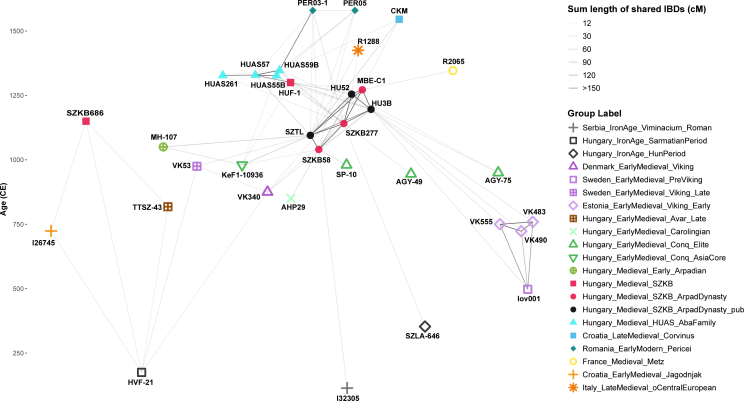
The IBD network of individuals who share at least 12cM length of cumulative IBD segments with the R-ARP-positive individuals and the fetus The points have been arranged according to published radiocarbon or inferred dating on the *Y* axis.

Although SZKB686 belongs to haplogroup R-ARP, it shows no direct IBD connection to any Árpád Dynasty individuals either with correctKin or with IBD. This is consistent with the Y chromosome analysis, which places SZKB686 in an older sub-branch of the R-ARP lineage, lacking recent genealogical ties to the dynasty members. Interestingly, the SZKB686 individual carrying the most ancestral R-ARP Hg, yielded radiocarbon dates overlapping with those of known Árpád Dynasty members. This suggests that SZKB686, who likely lived contemporaneously with the medieval Hungarian kings, is not an ancestor but rather a descendant of the common ancestor carrying the R-ARP Hg. The burial of this individual in the Royal Basilica may reflect a recognized distant kinship with the royal lineage. Despite the fact that with correctKin we could not detect close kinship among the Árpáds and HUF-1, the unidentified female fetus buried adjacent to Béla III, the IBD analysis revealed its genetic relatedness to multiple members of the Árpád Dynasty (Figures 4 and S6; Table S6), including Béla II, Béla III, and HU52, as well as to a member of the Aba family (HUAS57). Based on these data, and in the absence of genomic information from additional rulers and their relatives, the identity of the fetus remains unknown for now (Figure S1).

**Figure 5:**
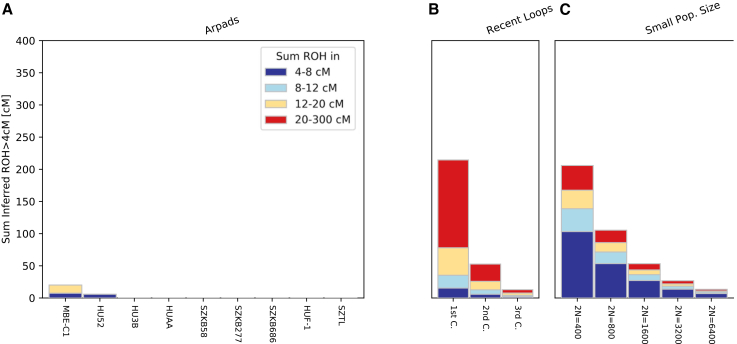
Runs of homozygosity (ROH) analysis Sum of inferred ROH > 4 cM across Árpád individuals compared to reference expectations for parental relatedness and long-term small population size. (A) Árpád individuals show low ROH levels indicating the absence of close-kin unions. (B) Expected ROH segment distribution resulting from recent consanguinity in the family history. (C) Expected ROH segment distribution resulting from small effective population size (2 N).

To exclude the possibility of recent inbreeding in the royal family, runs of homozygosity (ROH) analysis was performed (Figure 5). The results showed that none of the examined members of the royal family had long ROH segments. However, the presence of low level short ROH fragments in HU52 and MBE-C1 nicely corroborates the findings of the IBD analysis which indicated—through unexpected level of IBD sharing—distant consanguinities in their family histories.

### Distant and dynastical connections of the Árpáds

Besides determining familial kinship connections, IBD analysis is suitable to detect more distant relationship. This can extend to up to 8–9^th^ degree or even farther genetic connections due to the randomness of recombination and segregation. We wanted to see the dynastic connections of the Árpáds and their other distant relatives, so we introduced an extended medieval dataset into the IBD analysis including conquering Hungarians, medieval Hungarian noble families such as the Abas, Corvins, and Báthorys, as well as broadly contemporaneous European individuals (Table S6).

In the IBD network (Figure 4), the Árpád Dynasty individuals form a tight cluster. The core Árpád Dynasty members generally show genetic connections with conquering Hungarian elite individuals, including those referred to as the Conqueror Asia Core, such as KEF1-10936 and LB-1432,^24^ as expected.

They are also connected to previously identified members of Hungarian noble families.^25^^,^^26^^,^^27^ Multiple members of the Aba Dynasty (HUAS59B, HUAS57, HUAS55B, and HUAS261) show detectable relatedness to several Árpád Dynasty members, as previously demonstrated by Varga et al.^25^ Similarly, kinship links are observed with the Corvin and Báthory families (CKM, PER03-1, and PER05). The Árpáds’ IBD connections to the Abas and Christopher Corvinus confirm historical records: King Sámuel Aba, the eponymous ancestor of the Aba family, married a sister of Stephen I,^19^^,^^20^ while Beatrice de Frangepan, mother of Christopher Corvinus, descended from King Stephen V.^28^ The kinship with the Báthory family is also documented in Hungarian historiography.^29^

We also identified shared IBD fragments between an Early Medieval Danish Viking individual and Béla II, Béla III, and HU52 (Table S6). These Nordic/Viking connections within the Árpád lineage can be explained by the genetic contribution of multiple Rurikid queen consorts in the dynasty (Figure S4). In addition, Béla III also shows private connections to members of an Early Medieval Viking Age family from Estonia (VK483, VK490, VK555),^30^ and a Pre-Viking Era individual from Sweden (lov001).^31^ Notably, he shares the same IBD segment with two of the Estonian Vikings (VK483 and VK490) and the Swedish Pre-Viking individual (data not shown). Béla III’s genetic connection with Northern European individuals corresponds to his ancestry recorded in historical sources: his mother, Euphrosyne of Kiev (1130–1193), was a descendant of the Viking-origin Rurik dynasty.^16^

SZKB686 shares IBD fragments with an Early Medieval Croatian (I26745),^32^ an Avar (TTSZ-43),^24^ as well as an Iron Age Sarmatian (HVF-21) individual,^23^ which indicate a significant local Carpathian basin ancestry for him.

### Principal component analysis

**Figure 6:**
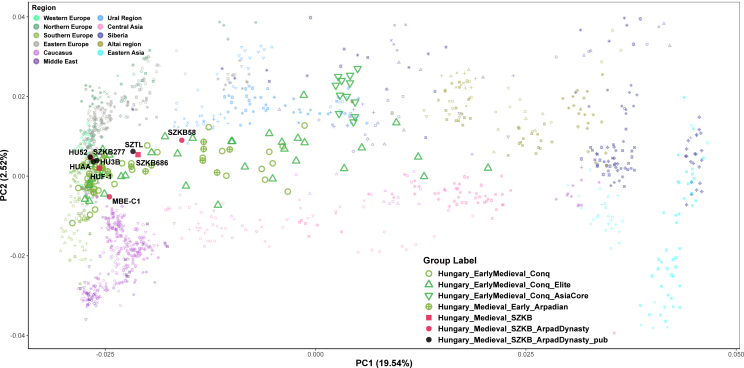
The Árpád Dynasty-related individuals projected over onto a Eurasian PCA together with published Hungarian Conquest Period individuals Transparent symbols represent the background containing 1,381 modern individuals (Table S8). Red symbols denote the Árpád Dynasty relatives described in this article while black symbols denote previously published relatives. Green symbols denote Hungarian Conquest Period individuals published in Maróti.^24^ These were included to represent the trend of genomic composition observed during this era of the Carpathian Basin.

The PCA results projecting the additional samples onto a standard Eurasian background^24^ reveal that two of the samples, SZKB58 and SZKB686, are notably shifted eastward from the main European cluster (Figure 6) (Table S8). Their positions fall within the genetic cline characteristic of the 10th-century conquering Hungarians. This eastward displacement is attributable to the presence of eastern genomic segments typical of that population. Moreover, the PCA placement of the identified Árpád Dynasty members aligns closely with their genealogical positions in Figure 1. SZKB58, the earliest member of the known R-ARP individuals, harbors the highest proportion of eastern genomic components. St. Ladislaus, at one or two generations distance, carries fewer eastern SNPs, while the later family members, Béla II the Blind (SZKB277), Béla III (HU3B), HU52 and HUF-1 cluster within the European genetic space, exhibiting minimal eastern ancestry. These patterns clearly demonstrate that the original eastern conquering Hungarian genetic heritage of the dynasty was progressively diluted over generations through European dynastic marriages. Béla, Duke of Macsó (MBE-C1) exhibits a slight southern European or near eastern affinity, which may be explained by his grandmother being Maria Laskarina, a Byzantine princess and the daughter of the Nicaean emperor Theodor Laskaris.^20^

### aHISplex analysis

To determine the most plausible phenotypic traits of the studied individuals we applied the aHISplex method^33^, which combines genome imputation with the HIrisPlex-S system. The phenotypic characteristics predicted by aHISplex are summarized in Tables 4 and S9. Most individuals are predicted to have brown eyes, brown hair, and an intermediate skin complexion. Notable exceptions include St. Ladislaus, who likely had blue eyes and light skin; Béla II, who had blue eyes; Béla, Duke of Macsó and HUF-1, both of whom had dark blond hair; and SZKB686, who exhibited an intermediate to dark complexion.

**Table 4:** Predicted eye, hair, and skin color of the studied individuals, using the aHISplex system

Identity	Sample ID	Eye	Hair	Skin	Publications
St. Ladislaus	SZTL	blue	brown/dark-brown	pale	previously published^8^
Béla III	HU3B	brown	dark brown/black	intermediate	previously published^7^^,^^8^^,^^11^
Anna of Antioch	HUAA	brown	dark-brown/black	intermediate	previously published^7^^,^^8^^,^^11^
Prince Andrew?	HU52	brown	brown/dark-brown	intermediate	previously published^7^^,^^8^^,^^11^
Béla, Duke of Macsó	MBE-C1	brown	dark-blond/brown	intermediate	this paper
Béla II (The Blind)	SZKB277	blue	brown/dark-brown	intermediate	this paper
Vazul?	SZKB58	brown	dark-brown/black	intermediate/lighter	this paper
Unknown male	SZKB686	brown	brown/dark-brown	intermediate/darker	this paper
Unknown female fetus	HUF-1	brown	dark-blond/brown	intermediate/lighter	this paper

Unfortunately, due to the scarcity of written records and the unreliability of drawings of the kings’ physical characteristics in manuscripts written after their death, we could not match these characteristics to specific rulers. We believe that as the methodology of predicting physical characteristics from genetic data improves, a finer description of the royals will emerge.

Our objective was to identify and characterize additional members of the Árpád Dynasty among the remains originally laid to rest in and around the Székesfehérvár Basilica. Based on prior and the current work, of the eight Árpád Dynasty kings buried at Székesfehérvár, only Béla II and Béla III could be conclusively identified, leaving six kings still awaiting identification. Prior work identified a grandson of Béla III, likely Andrew of Halych, and the current work identified a second degree relative of Ladislaus I, whom we could not positively identify. It is of interest that this skeleton had a pronounced malformation of the skull attributed to premature closing of the sagittal suture of the skull. The only member of the Árpád Dynasty who has been described as physically malformed was Coloman I, the nephew of St. Ladislaus. However, fourth degree relationship between SZKB58 and Béla II (SZK277) is inconsistent with this possibility. Similarly, coins found adjacent to the remains, four coins from St. Stephen’s time, and one coin from the time of King Péter Orseolo (1038–1041; 1044–1046) are also indicating an earlier burial time. The skull is very well preserved, indicating that it was protected from the elements, and this could be related to the physical location or depth of the tomb, considering the high ground water level at the site. The still missing kings may be among the samples that did not yield sufficient DNA for reliable Y-haplogroup determination, or among the remains without associated skulls, which were at this time excluded from this study. It is also possible that additional remains are yet to be discovered, as the westernmost section of the Basilica lies beneath the Bishop’s Palace and has not been excavated. Clearly, a more complete understanding of the Árpád Dynasty’s family structure will require further research.

The Árpád Dynasty’s R-ARP haplogroup is part of the R1a-Z93 branch, characteristic of Middle–Late Bronze Age Steppe groups like the Sintashta and Srubnaya, later present in eastern Scythians and both Steppe and Romanian Sarmatians.^23^^,^^34^^,^^35^^,^^36^^,^^37^ It also appears in Xiongnu remains from Mongolia, some showing high Y-STR matches with Béla III.^38^^,^^39^ Within R-Z93, the dynasty belongs to the R-Z2123 lineage, which based on our prior work on minorities of the ex-Soviet Union are most prominent in the Urals and in the Caucasus. The royal subbranch (R-Y20746 > R-SUR51) is found only among Southern Ural Bashkirs and Árpád Dynasty members, suggesting a unique paternal link to this population.^8^^,^^37^ Based on the time to the most recent common ancestor, the separation of the Árpád Dynasty lineage and the Bashkirs happened around mid-first millennium AD.^8^^,^^40^ Based on the currently known R-ARP positive individuals it was possible to break up the R-ARP haplogroup into four sub-haplogroups, R-ARP5, R-ARP1, R-ARP11, R-ARP10. This allows us to put the R-ARP SNPs into chronological order: The R-ARP5 SNPs (ARP2, ARP3, ARP5, ARP6, ARP8, ARP9) appeared earlier than the R-ARP1 SNPs (ARP1, ARP4, ARP7) and the R-ARP11 SNP (ARP11) appeared after these and before the R-ARP10 SNP (ARP10). The temporal resolution of the order of appearance of the 11 SNPs of the R-ARP haplogroup will be helpful to assign historical dates to the branching of Árpád Dynasty members yet to be discovered.

A further important finding of our work is that we have confirmed the Rurikid haplogroup previously described only in one Rurikid individual through the Y-haplogroup analysis of a second Rurikid prince,^12^ Béla, Duke of Macsó and that we also confirmed his relatedness to the Árpád Dynasty using kinship and IBD analysis. As additional data emerge from the genetic analysis of other European dynasties connected to the Árpáds, our conclusions can be refined, providing a stronger foundation for reconstructing the history of medieval Europe.

### Limitations of the study

This study reports only the Árpád-lineage carriers identified within the Székesfehérvár ossuary; the full population-genetic analysis of the >400 shotgun-sequenced individuals (>600 screened) is the subject of a companion preprint, “Genomic landscape of the medieval Hungarian elite from the Székesfehérvár royal necropolis” (bioRxiv: https://doi.org/10.64898/2026.04.10.717699 ENA: PRJEB111039). The companion analysis confirms that no additional Árpádian Y chromosome carriers and no further close kin of the dynasty—male or female—are present in the dataset.

Several individual findings retain residual uncertainty. The precise identity of SZKB58, a second-degree relative of St. Ladislaus, cannot be resolved from the available evidence. Radiocarbon dating of SZKB277 produced inconsistent results between two independent laboratories, with one returning a date over a century earlier than the period attributable to King Béla II; we treat the radiocarbon discrepancy as unresolved while noting that the genetic identification (second-degree relative of Béla III, third-degree of St. Ladislaus) is unambiguous. Queen Anna of Antioch (HUAA) could not be imputed because of low genome coverage and was therefore excluded from IBD analyses.

Finally, of the eight Árpád Dynasty kings interred at Székesfehérvár, only two (Béla II and Béla III) are now genetically confirmed; six remain unidentified. They may be among samples that did not yield sufficient endogenous DNA, among remains without an associated skull (excluded *a priori* from this study), or—given that the westernmost section of the basilica beneath the Bishop’s Palace has not been excavated—among remains yet to be recovered.

## Resource availability

### Lead contact

Further information and requests for resources should be directed to and will be fulfilled by the lead contact, Péter L. Nagy (plnagy@praxisgenomics.com).

### Materials availability

This study did not generate new unique reagents.

### Data and code availability

• All raw whole-genome sequencing data have been deposited at the European Nucleotide Archive under accession ENA: PRJEB96153 and are publicly available. Accession numbers are listed in the key resources table.
• This paper does not report original code; all software used is publicly available and cited in the STAR Methods.
• Any additional information required to reanalyze the data reported in this paper is available from the lead contact upon request.

## Acknowledgments

The authors express their gratitude to Péter Erdő Cardinal, Archbishop of Esztergom-Budapest for the permission to exhume the human remains from the Matthias Church in Budapest. We are grateful to Diocesan Bishop András Veres and Commissary Ferenc Reisner from the Diocese of Győr to enable the accession of the Ladislaus I bone material. We also like to thank Gábor Horváth-Lugossy and the leadership of the Szent István Museum of Székesfehérvár, Kovács Loránd Olivér, and the mayor of Székesfehérvár Cser-Palkovics András, for providing the conditions that allowed access to the ossuary of the National Memorial in Székesfehérvár. This work was supported by the House of Árpád Program (2018–2023), Scientific Subproject V.1 ("Anthropological-Genetic portrayal of Hungarians in the Árpadian Age"), grant to T.T., and grant no. VI/1878/2020 to E.N. The funders had no role in study design, data collection and analysis, the decision to publish, or the preparation of the manuscript.

## Author contributions

B.K. performed laboratory experiments and drafting of the manuscript; J.O. performed laboratory experiments and drafting of the manuscript; Z.M. carried out bioinformatics processing and data analysis; O.S. performed laboratory experiments and contributed to data analysis, visualization, and manuscript writing; N.R. contributed to the data analysis; M.F.N. contributed to the bioinformatical data analysis; A.G. assisted with experimental execution; K.M. performed the majority of the laboratory work; B.T. curated archaeological and anthropological data; L.K. curated anthropological data; B.H. advised on the historical context; B.K. provided insights to historical context; Z.S.B. helped the bone material collection and contributed to anthropological analyses; Z.S.Z. provided illustration of the basilica; E.N. contributed to experimental planning; T.T. participated in experimental planning, organizing and manuscript writing; M.K. organized the project and contributed to experimental design; P.L.N. inception of the project, procuring founding, study execution, data generation and analysis, and writing of the article; G.I.B.V. organized and oversaw the experiments and their execution and contributed to the critical review and writing of the article. All authors reviewed and approved the final manuscript.

## Declaration of interests

Peter Lajos Nagy is the founder and owner of Praxis Genomics LLC.

## Declaration of generative AI and AI-assisted technologies in the writing process

During the preparation of this manuscript, the authors used Claude (Anthropic) to audit the document format against the iScience Final File Requirements and to perform structural editing. The tool was not used to generate, paraphrase, or write any scientific content. After using this tool, the authors reviewed and edited the document as needed and take full responsibility for the content of the publication.

## STAR★Methods

### Key resources table

**Table:** 

REAGENT or RESOURCE	SOURCE	IDENTIFIER
**Biological samples**		

Skeletal remains SZKB58 (R-ARP+, 2nd-degree relative of St. Ladislaus)	Ossuary, Royal Basilica of Székesfehérvár; King Saint Stephen Museum	ID 95.4.30; Éry^11^ V/8
Skeletal remains SZKB277 (King Béla II)	Ossuary, Royal Basilica of Székesfehérvár; King Saint Stephen Museum	ID 93.2.40; Éry^11^ II/77
Skeletal remains SZKB686 (R-ARP+ unidentified)	Ossuary, Royal Basilica of Székesfehérvár; King Saint Stephen Museum	ID 93.1.17; Éry^11^ VI/86
Skeletal remains MBE-C1 (Béla, Duke of Macsó)	Hungarian Natural History Museum, Department of Anthropology	Margaret Island Monastery (1915 excavation); Hajdú et al.^12^
Skeletal remains HUF-1 (fetus, tomb adjacent to Béla III)	Reinterred Matthias Church, Buda; this study	N/A
Previously published Y-haplogroup R-ARP genomes: HU3B (Béla III), HU52, HUAA, SZTL (St. Ladislaus)	Wang et al.^38^; Varga et al.^10^	See respective publications

**Chemicals, peptides, recombinant proteins**		

Proteinase K	Various	N/A
EDTA (0.45 M)	Various	N/A
Triton X-100	Various	N/A
Guanidine hydrochloride	Various	N/A
USER enzyme	New England Biolabs	Cat# M5505
Uracil Glycosylase Inhibitor (UGI)	New England Biolabs	Cat# M0281
T4 polynucleotide kinase	Thermo Scientific	N/A
T4 DNA polymerase	Thermo Scientific	N/A
T4 DNA ligase	Thermo Scientific	N/A
Tango Buffer (10*X*)	Thermo Scientific	N/A
ThermoPol® reaction buffer	New England Biolabs	Cat# B9004
Bst polymerase, large fragment	New England Biolabs	Cat# M0275
Bovine serum albumin (BSA)	Various	N/A

**Critical commercial assays**		

MinElute PCR Purification Kit/DNA purification columns	QIAGEN	Cat# 28006
Accuprime Pfx Supermix	ThermoFisher Scientific	Cat# 12344040
Qubit fluorometric quantification system	ThermoFisher Scientific	Cat# Q33231
TapeStation 2200 system	Agilent Technologies	G2964AA
iSeq 100 i1 Reagent v2 (cartridge + flow cell)	Illumina	Cat# 20031374

**Deposited data**		

Whole-genome sequencing data, this study	European Nucleotide Archive (ENA)	ENA: PRJEB96153
Companion population genetics dataset (embargoed until publication)	European Nucleotide Archive (ENA)	ENA: PRJEB111039
Companion preprint: Genomic landscape of the medieval Hungarian elite from the Székesfehérvár royal necropolis	bioRxiv	https://doi.org/10.64898/2026.04.10.717699
Reference human genome (GRCh37/hg19, NCBI build 37)	Genome Reference Consortium	https://www.ncbi.nlm.nih.gov/grc/human
Allen Ancient DNA Resource (AADR v42.4)	Reich Lab, Harvard Medical School	https://reich.hms.harvard.edu/allen-ancient-dna-resource-aadr-downloadable-genotypes-present-day-and-ancient-dna-data
yfull Y chromosome database	yfull	https://www.yfull.com/
ISOGG Y-DNA haplogroup repository	International Society of Genetic Genealogy	https://isogg.org/

**Oligonucleotides**		

Illumina P5/P7 universal adapter molecules	Custom synthesized (Sigma-Aldrich)	https://www.sigmaaldrich.com/

**Software and algorithms**		

cutadapt	Martin^41^	https://cutadapt.readthedocs.io/
FastQC	Andrews^42^	https://www.bioinformatics.babraham.ac.uk/projects/fastqc/
BWA v0.7.17-r1188	Li and Durbin^43^	http://bio-bwa.sourceforge.net/
SAMtools v1.12	Li et al.^44^	http://www.htslib.org/
Picard Tools	Broad Institute^45^	https://github.com/broadinstitute/picard
ATLAS	Link et al.^46^	https://bitbucket.org/wegmannlab/atlas/
ANGSD	Korneliussen et al.^47^	https://github.com/ANGSD/angsd
mapDamage 2.0	Jónsson et al.^48^	https://ginolhac.github.io/mapDamage/
Schmutzi	Renaud et al.^49^	https://github.com/grenaud/schmutzi
Mosdepth	Pedersen and Quinlan^50^	https://github.com/brentp/mosdepth
HaploGrep 2	Weissensteiner et al.^51^	https://haplogrep.i-med.ac.at/
Yleaf	Ralf et al.^52^	https://github.com/genid/Yleaf
correctKin	Nyerki et al.^53^	https://github.com/zmaroti/correctKin
smartpca (EIGENSOFT)	Reich Lab, Harvard Medical School	https://github.com/chrchang/eigensoft
GLIMPSE2 v2.0.0	Rubinacci et al.^54^	https://odelaneau.github.io/GLIMPSE/
ancIBD v0.5	Ringbauer et al.^21^	https://github.com/hringbauer/ancIBD
scoreFilterIBD	Schütz et al.^23^	https://github.com/zmaroti/scoreFilterIBD
ibdsim2 (R package)	Vigeland^22^	https://cran.r-project.org/web/packages/ibdsim2/
pedsuite (R package collection)	Vigeland^22^	https://cran.r-project.org/web/packages/pedsuite/
HIrisPlex-S/aHISplex	Maróti et al.^33^	See STAR Methods
OxCal v4.4.4	Bronk Ramsey, University of Oxford	https://c14.arch.ox.ac.uk/oxcal/
Python v3.6.8	Python Software Foundation^55^	https://www.python.org/
R v4.1.0	R Core Team	https://www.r-project.org/

**Other**		

Illumina iSeq 100 sequencer	Illumina	Cat# 20021535
Illumina NovaSeq 6000 sequencer	Illumina	Cat# 20012850
Dremel® 3000 multifunctional hand drill	Dremel	N/A
VWR™ Star-Beater ball grinder	VWR	N/A

### Experimental model and study participant details

#### Ethical approval

No ethical approval was required since the samples studied are all ancient remains.

#### Description of the samples studied

The human remains of King Béla III (HU3B), Queen Anna of Antioch (HUAA) and their grandson, the presumed Prince Andrew, son of Andrew II (HU52), now rest in the Matthias Church and have been previously studied.^8^^,^^9^ The samples of more than 600 individuals from the ossuary of the Royal Basilica were processed with authorization from the Diocese of Székesfehérvár, the city of Székesfehérvár, and the King Saint Stephen Museum, under whose supervision these remains are held. The full population genetic analysis of these remains is being written up in a separate publication. The skeletal remains of Béla, Duke of Macsó were provided by the Hungarian Natural History Museum, Department of Anthropology, Budapest. The sampling of remains from the ossuary of the Székesfehérvár Basilica was carried out following specific criteria. Priority was given to skeletons with a preserved skull. When the petrous bone was available, it was sampled by drilling; otherwise, an intact tooth was extracted from the mandible as an alternative source of DNA. In this paper, we discuss only the remains that can be associated with the Árpád Dynasty (Figures 1 and 2). The results of their anthropological examination are summarized from the description provided by Érdy^5^ and Éry et al.^11^

**Figure 7:**
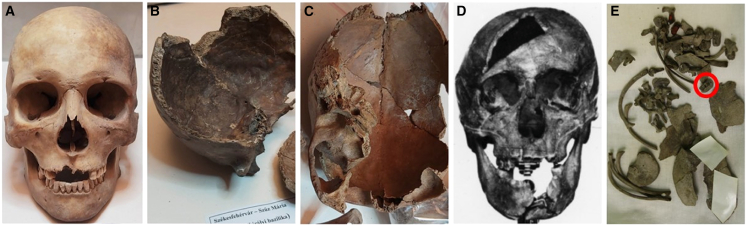
Remains of the individuals genetically analyzed in this study (A) SZKB58; (B) SZKB277; (C) SZKB686; (D) MBE-C1; (E) HUF-1.

SZKB58 (Figure 7A) (ID number: 95.4.30; serial number in Éry et al. 2008: V/8) was a well-preserved skeleton, identified as an adult male (43–47 years old) based on traits observed on both the skull and postcranial skeleton. The estimated stature of the individual was 175 cm, calculated from the average of values derived from the humerus, radius, femur, and tibia. Pathological advanced ossification of the sagittal suture was observed on the skull which slightly affected the morphology of the parietal bones. Four coins from St. Stephen’s time, and one coin from the time of King Péter Orseolo (1038–1041; 1044–1046) were found near SZKB58. The remains of SZKB686 (Figure 7C) (ID number: 93.1.17; serial number in Éry et al. 2008: VI/86) belonged to a sub-adult. The individual was identified as a juvenile (17–19 years old), based on the degree of epiphyseal fusion. Due to the individual’s young age, biological sex and stature could not be determined. No pathological changes or developmental disorders were detected on the preserved remains. SZKB277 (Figure 7B) (ID number: 93.2.40.; serial number in Éry et al. 2008: II/77) was identified as an adult male (30–60 years old). However, only a limited number of traits on the skull were available for determining biological sex and estimating age at death. No pathological changes or disorders were observed on the preserved skeletal remains. The skeletal remains of MBE-C1 (Figure 7D) were discovered at the Margaret Island Monastery. The initial anthropological assessment was conducted by Buzár and Bernert.^14^ In a subsequent interdisciplinary study, Hajdú et al. performed anthropological, radiocarbon, and archaeogenetic analyses.^12^ The combined results consistently indicate that the remains belonged to a young adult male, estimated to be approximately 19.8–30.9 years old. Multiple perimortem sharp-force injuries observed on the skull and postcranial skeleton – including three major sword blows to the cranium and cuts to the face and jaw – suggest deliberate fatal violence and possible mutilation. The combined anthropological, historical, and genetic evidence supports the identification of the remains as those of Béla, Duke of Macsó. HUF-1 (Figure 7E) was identified as a fetal skeleton. It was discovered on the left side of the pelvis of a pregnant woman who died between the ages of 20 and 30 years (Figure S1). The height of the frontal bone is 50 mm, and its width is 45 mm. The length of the iliac crest measures 28 mm, while its width is 27 mm. These measurements are typical for a fetus aged 9 to 9.5 lunar months.^11^ Details regarding the samples, including their identifiers, key characteristics, and sampling sites, are presented in Table S2.

#### Biological sex and the cohort sex distribution

Biological sex for every individual was inferred from sequencing data using the X/Y read-count method of Skoglund et al.^56^ and is reported in Table S2. The Árpád Dynasty is defined paternally by the R-ARP Y chromosome haplogroup, so the male-to-female ratio of Árpád-lineage members analyzed here is biased toward males by definition rather than by sample selection. The only previously identified Árpád-related female (Queen Anna of Antioch, HUAA) was excluded from IBD analyses owing to low genome coverage, and the small sample size precludes a formal test of sex effects on the kinship or IBD measures reported.

### Method details

#### Radiocarbon analysis

Radiocarbon analyses were conducted in two independent laboratories to validate the dating of samples SZKB58, SZKB277, and SZKB686. The samples were analyzed using accelerator mass spectrometry (AMS) at two institutions: the AMS laboratory of the Institute for Nuclear Research, Hungarian Academy of Sciences, Debrecen, Hungary (for technical details on sample preparation and measurement, see papers by Molnár and colleagues,^57^^,^^58^ and the Center for Applied Isotope Studies of the University of Georgia (technical details available at: https://cais.uga.edu/facilities/radiocarbon-ams-facility/). The conventional radiocarbon data were calibrated using the OxCal 4.4.4 software (https://c14.arch.ox.ac.uk/oxcal/OxCal.html, date of calibration: 08.08.2025) with IntCal20 settings.

#### DNA extraction and library construction

All steps of sampling, DNA extraction and library preparation were carried out as described in the Supplementary materials of Varga et al., 2023,^10^ in the joint, dedicated ancient DNA laboratory of the Department of Archaeogenetics, Institute of Hungarian Research and the Department of Genetics, University of Szeged. Bone pieces were prepared using a Dremel® 3000 multifunctional hand drill and powdered with a VWR™ Star-Beater ball grinder. DNA extraction was carried out by cleaning and soaking the whole teeth or 200 mg bone powder in digestion buffer (0.45 M EDTA, 250 μg/mL Proteinase K, 0.1% Triton X-100) for 72 h on 48 °C, than binding the DNA on Qiagen™ MinElute DNA purification columns with freshly prepared binding buffer (5 M Guanidine hydrochloride, 90 mM Sodium acetate, 40% Isopropanol and 0.05% Tween 20). Elution was carried out with a standard TE buffer (1 mM EDTA, 10 mM TRIS-HCl).

We prepared double-stranded DNA libraries according to the protocol described in Meyer and Kircher^59^ with minor modifications. We applied partial UDG treatment to counteract the effects of extensive postmortem damage (PMD). The reaction mix was prepared as described in Rohland et al.,^60^ containing 1*X* Tango Buffer (Thermo Scientific™), 100 μM dNTPs, 1 mM ATP and 0.03 U/μL USER enzyme with 30 μL of sample DNA. We incubated the samples for 30 min, then stopped the reaction with Uracil Glycosylase Inhibitor (UGI). The samples were prepared for adapter ligation with blunt-end repair using a mixture of T4 polynucleotide kinase (0.5 U/μL) and T4 DNA polymerase (0.1 U/μL) and incubated for 20 min at 25°C and 15°C. Following this, the samples were purified on Qiagen™ MinElute columns and eluted in 20 μL Elution Buffer (EB). Adapter ligation was carried out according to Meyer et al.^59^ We used universal P5 and P7 adapter molecules in a mixture of 1*X* T4 DNA ligase buffer (Thermo Scientific™), 5% PEG-4000, 1.25 μM adapter mix and 0.125 U/μL T4 DNA ligase with 20 μL purified sample DNA. We incubated the samples for 30 min at 22 °C, followed by a second round of DNA purification on MinElute columns. Finally, we carried out an adapter fill-in reaction with 1*X* ThermoPol® reaction buffer (NEB®), 250 μM dNTPs and 0.3 U/μL Bst polymerase large fragment with 20 μL sample DNA to fill out the partially single-stranded adapters and correct any remaining nucleotide errors on one of the strands. We omitted preamplification and directly double indexed our libraries in a single PCR step with Accuprime™ Pfx Supermix (Invitrogen™), containing 10 mg/mL BSA and 200 nM indexing P5 and P7 primers, in the following cycles: 95 °C 5 min, 12 times 95 °C 15 s, 60 °C 30 s and 68 °C 3 s, followed by 5-min extension at 68 °C. The indexed libraries were purified on MinElute columns and eluted in 20 μL EB.

#### Low-coverage and high-coverage sequencing

The human DNA content of the libraries was evaluated using low-coverage paired-end (2 × 150) sequencing using the Illumina iSeq 100 sequencer (Illumina, Inc., San Diego, CA, USA, Catalog Number: 20021535) (Table S3A). Based on the prior shallow sequencing data, we selected only the eligible samples with suitable endogenous content for deep sequencing. Accordingly, only one library preparation was made for each sample. Depending on the endogenous DNA content, we performed a higher-coverage paired-end (2 × 100-150) sequencing at Praxis Genomics LLC (Atlanta, GA, USA) and iBioScience Kft. (Pécs, Hungary) on the NovaSeq6000 device (Illumina, Inc., USA, Catalog Number: 20012850). The QC metrics for each sample are described in Table S3.

#### Data analysis

##### Raw data handling, quality checking

The adapters of paired-end reads were trimmed with the Cutadapt software,^41^ and sequences shorter than 25 nucleotides were removed. Read quality was assessed with FastQC.^42^ The raw reads were aligned to GRCh37 (hs37d5) reference genome using the Burrows-Wheeler-Aligner (v 0.7.17) software, with the MEM command in paired mode, with default parameters and disabled reseeding.^43^ Only properly paired primary alignments with ≥90% identity to reference were considered in all downstream analyses to remove exogenous DNA. Samtools v1.1 was used for merging the sequences for different lanes, sorting, and indexing binary alignment map (BAM) files.^44^ PCR duplicates were marked using Picard Tools MarkDuplicates v 2.21.3.^45^ To randomly exclude overlapping portions of paired-end reads and to mitigate potential random pseudo-haploidization bias, we applied the mergeReads task with the options “updateQuality mergingMethod = keepRandomRead” from the ATLAS package.^46^ Single nucleotide polymorphisms (SNPs) were called using the ANGSD software package (version: 0.931–10-g09a0fc5)^47^ with the “-doHaploCall 1 -doCounts 1” options and restricting the genotyping with the “-sites” option to the genomic positions of the 1240 K panel.

Ancient DNA damage patterns were assessed using MapDamage 2.0^48^ (Table S3B). Mitochondrial genome contamination was estimated using the Schmutzi algorithm.^49^ Contamination for the male samples was assessed by the ANGSD X chromosome contamination estimation method,^61^ with the “-r X:5000000–154900000 -doCounts 1 -iCounts 1 -minMapQ 30 -minQ 20 -setMinDepth 2” options.

##### Genetic sex and haplogroup determination, kinship

Biological sex was assessed with the method described in.^56^ Fragment length of paired-end data and average genome coverages (all, X, Y, mitochondrial) were assessed by the ATLAS software package^46^ using the BAMDiagnostics task. Detailed coverage distributions of autosomal, X, Y chromosomes and mitochondrial DNA were calculated by the mosdepth software.^50^

Mitochondrial haplogroup (Mt Hg) determination was performed with the HaploGrep 2 (version 2.1.25) software,^51^ using the consensus endogen fasta files resulting from the Schmutzi Bayesian algorithm. The Y Hg assessment was performed with the Yleaf software tool,^52^ updated with the ISOGG2020 Y tree dataset, and was further refined using the current Family Tree dataset.

Kinship analysis was performed with correctKin.^53^ As reference population we applied the same database as in Varga et al. 2023.^10^

##### Principal component analysis - PCA

Smartpca was used to create a modern PCA background on which ancient samples could be projected, we used the modified modern Eurasian genome data, as described in Varga et al.^10^ (Table S8). All ancient genomes were projected on the modern background with the ‘‘lsqproject: YES and inbreed: YES’’ options.

##### IBD sharing analysis

For imputation, we used the GLIMPSE2 framework (version 2.0.0)^54^ using the 1 KG Phase 3 dataset common markers as reference. The reference dataset was normalized and multi allelic sites were split using bcftools (version 1.16–63-gc021478 using htslib 1.16–24-ge88e343) with the “norm –m –any” subcommand and filtered for biallelic SNPs with the “view –m 2 –M 2 –v snps” subcommand. The autosomal chromosomes of the human reference genome were divided into 580 genomic chunks using the GLIMPSE2_chunk tool with the “-sequential” option. As described in the GLIMPSE2 manuscript, we created the binary reference data with the GLIMPSE2_split_reference tool using the 580 genomic regions and the 1 KG biallelic SNP variants. In all downstream imputation analyses, we used only samples with >0.5*x* mean genome coverage of shotgun WGS data as recommended in the GLIMPSE2 manuscript. Furthermore, we excluded all samples with estimated MT contamination higher than 0.03 (based on the Schmutzi MT contamination analysis),^49^ as the higher MT-contaminated (0.06–0.12) samples had lower concordance according to our experiments using high coverage aDNA data. We used the ancIBD (version 0.5) python libraries with the Python 3.6.8 environment^55^ for IBD fragment analysis.^21^ Phased and imputed variants of experimental aDNA samples were post-filtered to include only the positions of the 1240 K AADR marker set and lifted to the hdf5 data format as described in the ancIBD manuscript.^21^ IBD fragments were identified with the default parameters recommended for aDNA analysis (emission model haploid_gl2, HMM model FiveStateScaled, and the p_col = ‘variants/RAF' option to use GLIMPSE2 reference AF data from the imputed variants). During the subsequent filtration of raw IBD segments, we deviated from the marker density threshold (≥220 SNPs/cM) used in the original ancIBD framework. We implemented a method described in Schütz et al.^23^ that uses marker informativity scores to dynamically mask and exclude genomic regions lacking sufficient power to detect true IBD segments.

##### Simulation of genealogical connections and analysis of IBD parameter distributions

To clarify the precise kinship between the R-ARP-carrying individual SZKB58, and St. Ladislaus, we applied an additional IBD-based approach. We generated simulated reference data in R using the ibdsim2 package taken from the pedsuite collection.^22^ We applied the “ibdsim” function with default parameters on different pedigrees to generated 500 independent kin relationships for direct and indirect lines of maternal and paternal descent between 1-5th degrees of relatedness as well as for the 2nd degree indirect non-conventional case of half-sibling (10000 simulations in total). The maternal and paternal distinction was introduced to ascertain the effect of the ∼1.6*x* higher recombination rate documented in females.^62^^,^^63^ Because the chromosomal lengths used in ibdsim2 differed slightly from the ones inferred in our samples by ancIBD,^21^ we rescaled the lengths of the simulated IBD fragments and applied a minimum length cutoff at 8 cM to produce comparable data structure. The simulation was verified by comparing the distribution of the simulated IBD fragment parameters with the distribution of real genealogical connections collected from our database of 2070 ancient published and unpublished genomes (Figure 3).

##### Runs of homozygosity analysis

We used hapROH^64^ with default parameters to detect runs of homozygosity (ROH) and test for consanguinity within the family.

##### Eye, hair and skin color prediction from DNA

The aHisPlex^33^ tool includes premade reference data needed for imputation by the GLIMPSE2 framework to impute all the haplotypes of the 11 genome regions containing the 41 eye/hair/skin associated markers used by the HIrisPlex-S system. By incorporating pre-made reference datasets for GLIMPSE2 to impute only the 11 genomic regions encompassing the 41 HIrisPlex-S markers, the software enabled efficient and accurate phenotyping of our ancient samples.

### Quantification and statistical analysis

All analyses were performed in Python (v3.6.8) and R (v4.1.0). Sample n: three Árpád-lineage carriers reported in this study (SZKB58, SZKB277, SZKB686), one fetus (HUF-1) and one Rurikid Árpád-relative (MBE-C1), analyzed together with five previously published R-ARP-positive Árpád individuals (HU3B, HU52, HUAA, SZTL, UVD). Per-sample QC, contamination, sex, and haplogroup calls are reported in Table S3. Kinship analyses (correctKin^53^) report point estimates of kinship coefficients with the standard errors output by the software (Table S5). IBD analyses (ancIBD21) were filtered using marker-informativity scores as described in Schütz et al.^23^ and applied with a minimum segment length cutoff of 8 cM. Simulated kinship-to-IBD distributions shown in Figure 3 were generated from 500 independent simulations per pedigree configuration across 20 pedigree configurations (10,000 simulations in total) using ibdsim2 22 from the pedsuite22 package collection. Runs of homozygosity (Figure 5) were detected with hapROH^64^ at a minimum segment length of 4 cM; per-individual values shown are the sum of inferred ROH segments. PCA (Figure 6) projected ancient samples onto a Eurasian background of 1,381 modern individuals (Table S8) using smartpca with the lsqproject: YES and inbreed: YES options. Sample sizes per analysis, error/dispersion measures (where applicable), and statistical details specific to each figure are reported in the corresponding figure legend.

## Supplemental information

Document S1. Figures S1–S7

Table S1. Summary of Yleaf, Y chromosome results Samples highlighted with green background are reported in this study. Haploid marker-based analysis of the other samples was previously published. They are included here because their autosomal genetic analysis was done together in this article with the samples reported here.

Table S2. Overview of samples, including nomenclature and key characteristics Samples highlighted with green background are reported in this study. Analysis of the other samples was previously published. They are included here because their autosomal genetic analysis was redone in this article together with the samples reported here.

Table S3. Per-sample sequencing quality control and postmortem damage metrics (A) Quality control (QC) metrics for all samples analyzed in this study; samples highlighted with green background are reported in this study, while analysis of the other samples was previously published and is included here because their autosomal genetic analysis was redone in this article together with the samples reported here. Note that the “Cemetery” and “Tomb characteristics” section refers to the original burial site of the remains; for present-day placement see Table S2. (B) Postmortem damage (PMD) metrics for all samples analyzed in this study; samples highlighted with green background are reported in this study, while analysis of the other samples was previously published and is included here because their autosomal genetic analysis was redone in this article together with the samples reported here.

Table S4. Summary of mitochondrial results Samples highlighted with green background are reported in this study. Haploid marker-based analysis of the other samples was previously published. They are included here for comparison.

Table S5. Kinship results calculated by correctKin

Table S6. Identity-by-descent (IBD) analyses of the Árpád Dynasty cohort against an ancient reference panel (A) Pairwise IBD-sharing results between every sample pair, including longest IBD segment length and cumulative IBD lengths at multiple cM thresholds. (B) Metadata for the ancient genomes used in the IBD analysis, including sample identifiers, AADR labels, dating, geographic origin, and coordinates.

Table S7. Runs of homozygosity results, based on the hapROH analysis

Table S8. List of modern background samples and data of ancient samples used for the Eurasian PCA

Table S9. Eye, hair, and skin color prediction using the aHISplex system
