# Supplementary material for: Genetic identification of Árpád Dynasty members from the ossuary of the Royal Basilica at Székesfehérvár
Source: iScience. 2026 Jun 12;29(7):116365. doi: 10.1016/j.isci.2026.116365 (PMC13276751; doi:10.1016/j.isci.2026.116365)
Supplement: Document S1. Figures S1–S7 [file mmc1.pdf]

iScience, Volume *29*

Supplemental information

Genetic identification of Árpád Dynasty members from the ossuary of the Royal Basilica at Székesfehérvár

Bence Kovács, Judit Olasz, Zoltán Maróti, Oszkár Schütz, Nicholas Rouse, Michael F. Nagy, Alexandra Gînguță, Kitti Maár, Balázs Tihanyi, Luca Kis, Balázs Holczmann, Balázs Kertész, Zoltán Szabó, Zsolt Bernert, Endre Neparáczki, Tibor Török, Miklós Kásler, Péter L. Nagy, and Gergely I.B. Varga

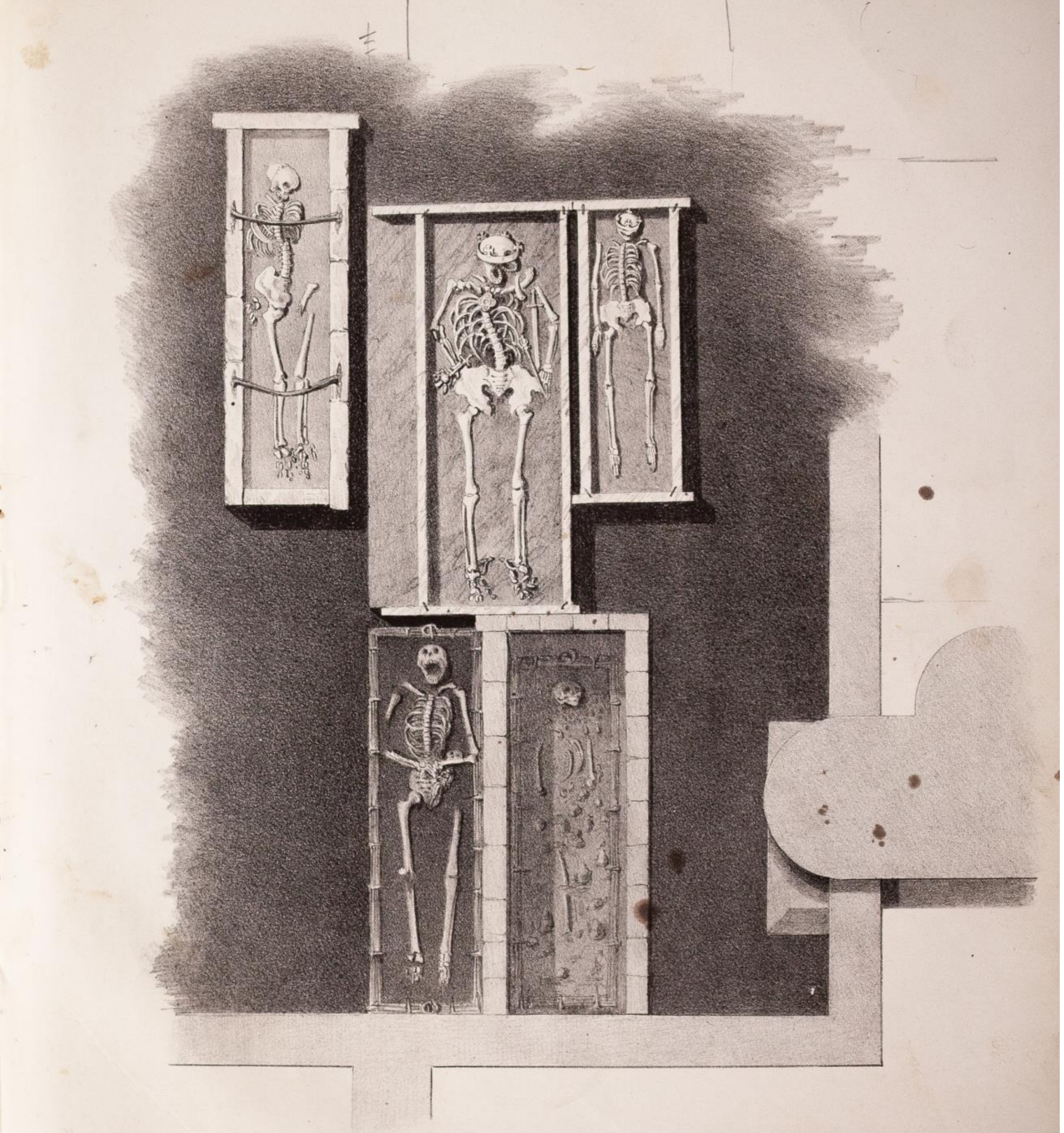

IIIik Béla király és nejének Székes-Fehérvárott talált síremlékei. (:Csontvázak koporsókban:)

Nyomt. Frank J. M. Pesten. 1853.

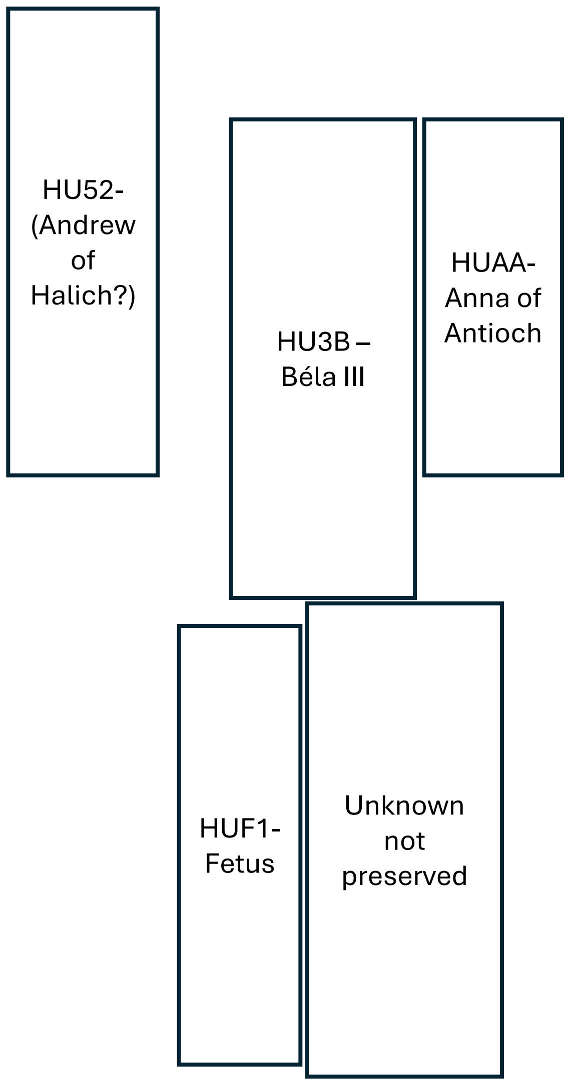

Translation: Burial site of King Béla III and his wife at Székesfehérvár. (Skeletons in coffins)

Érdy (1853)[5]

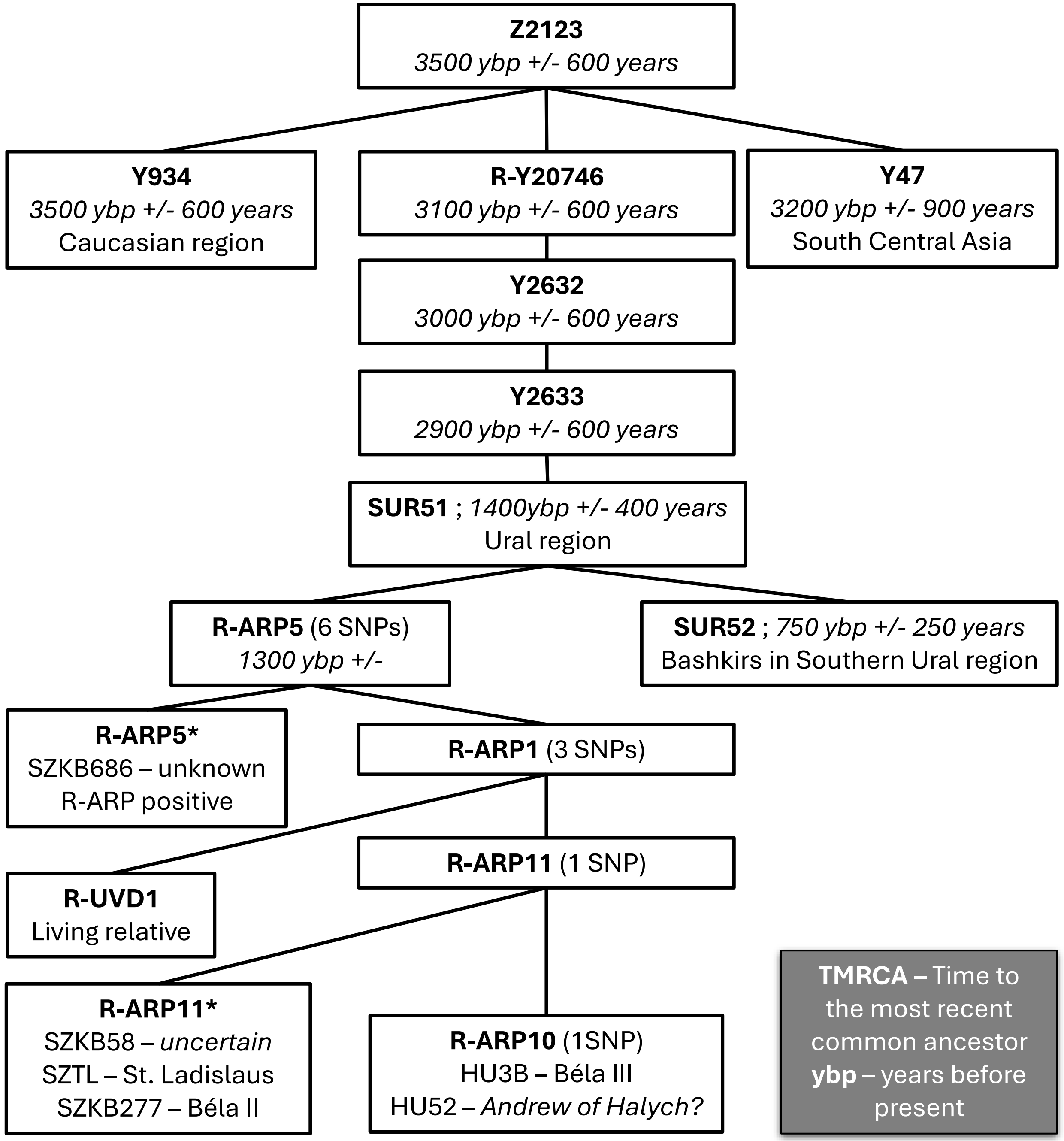
Z2123
3500 ybp +/- 600 years
Y934
3500 ybp +/- 600 years
Caucasian region
R-Y20746
3100 ybp +/- 600 years
Y47
3200 ybp +/- 900 years
South Central Asia
Y2632
3000 ybp +/- 600 years
Y2633
2900 ybp +/- 600 years
SUR51 ; 1400ybp +/- 400 years
Ural region
R-ARP5 (6 SNPs)
1300 ybp +/-
SUR52 ; 750 ybp +/- 250 years
Bashkirs in Southern Ural region
R-ARP5*
SZKB686 – unknown
R-ARP positive
R-ARP1 (3 SNPs)
R-UVD1
Living relative
R-ARP11 (1 SNP)
R-ARP11*
SZKB58 – uncertain
SZTL – St. Ladislaus
SZKB277 – Béla II
R-ARP10 (1SNP)
HU3B – Béla III
HU52 – Andrew of Halych?
TMRCA – Time to the most recent common ancestor
ybp – years before present

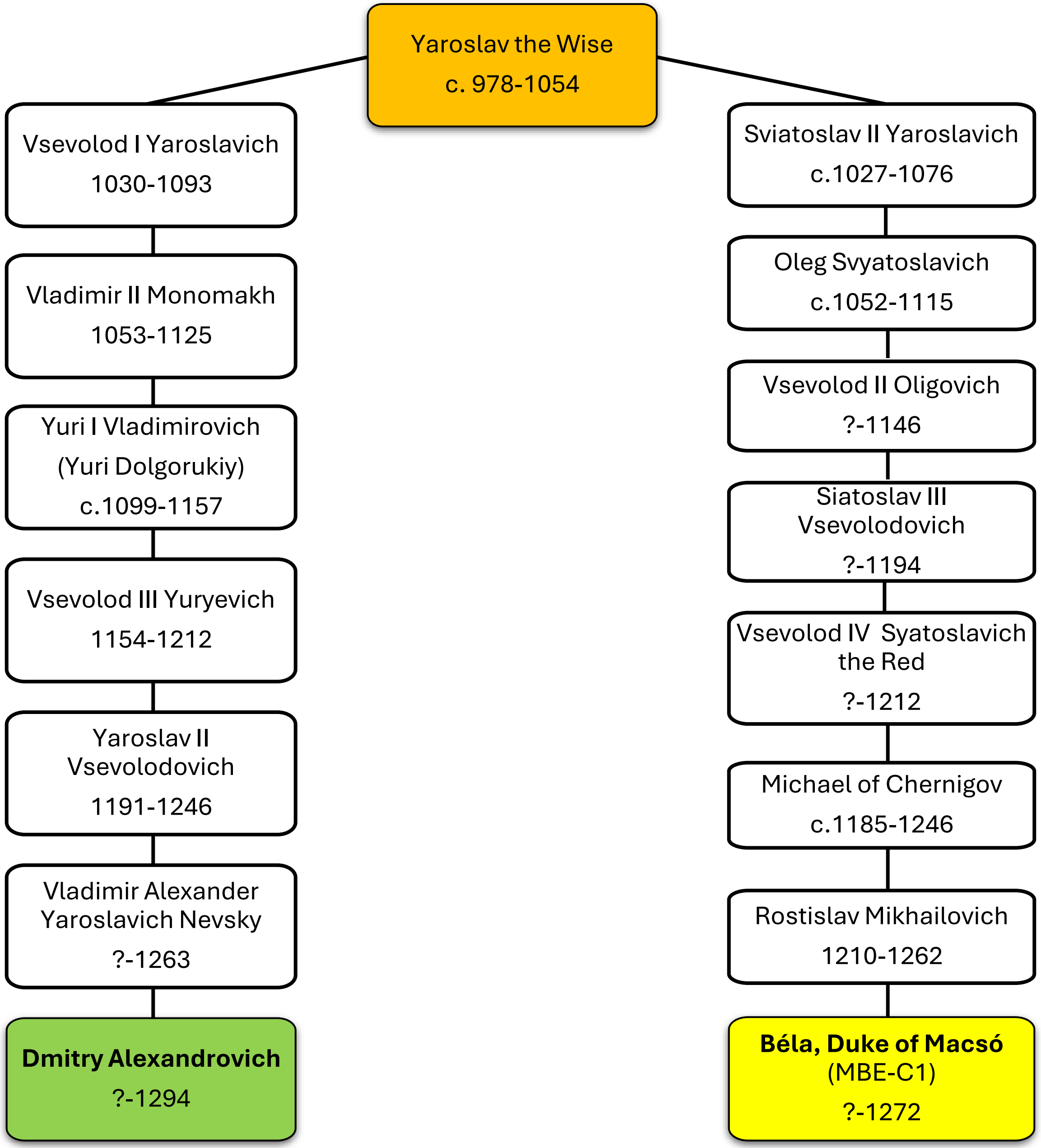

The paternal lineages of Dmitry Alexandrovich and Béla, Duke of Macsó

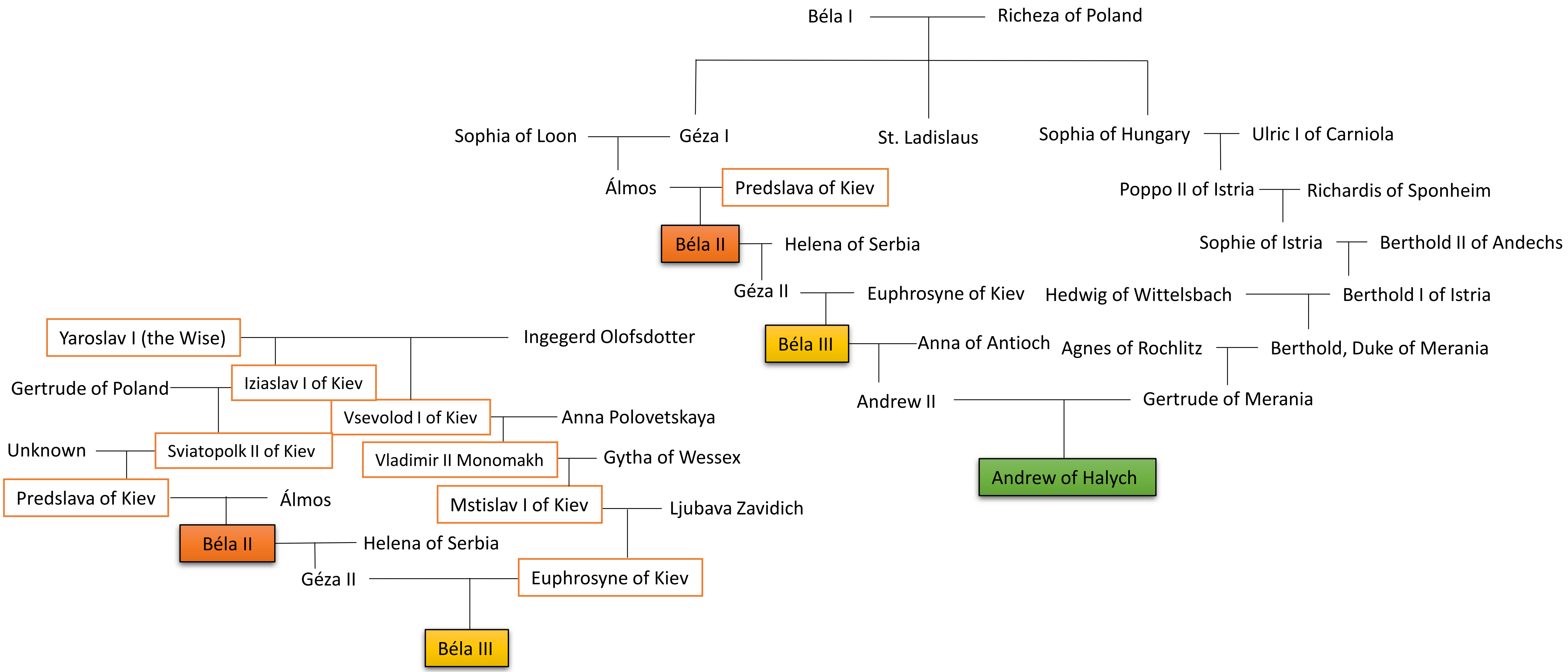
Béla I
Richeza of Poland
Sophia of Loon
Géza I
St. Ladislaus
Sophia of Hungary
Ulric I of Carniola
Álmos
Predslava of Kiev
Poppo II of Istria
Richardis of Sponheim
Béla II
Helena of Serbia
Sophie of Istria
Berthold II of Andechs
Géza II
Euphrosyne of Kiev
Hedwig of Wittelsbach
Berthold I of Istria
Yaroslav I (the Wise)
Ingegerd Olofsdotter
Béla III
Anna of Antioch
Agnes of Rochlitz
Berthold, Duke of Merania
Gertrude of Poland
Iziaslav I of Kiev
Vsevolod I of Kiev
Anna Polovetskaya
Andrew II
Gertrude of Merania
Unknown
Sviatopolk II of Kiev
Vladimir II Monomakh
Gytha of Wessex
Andrew of Halych
Predslava of Kiev
Álmos
Mstislav I of Kiev
Ljubava Zavidich
Béla II
Helena of Serbia
Géza II
Euphrosyne of Kiev
Béla III

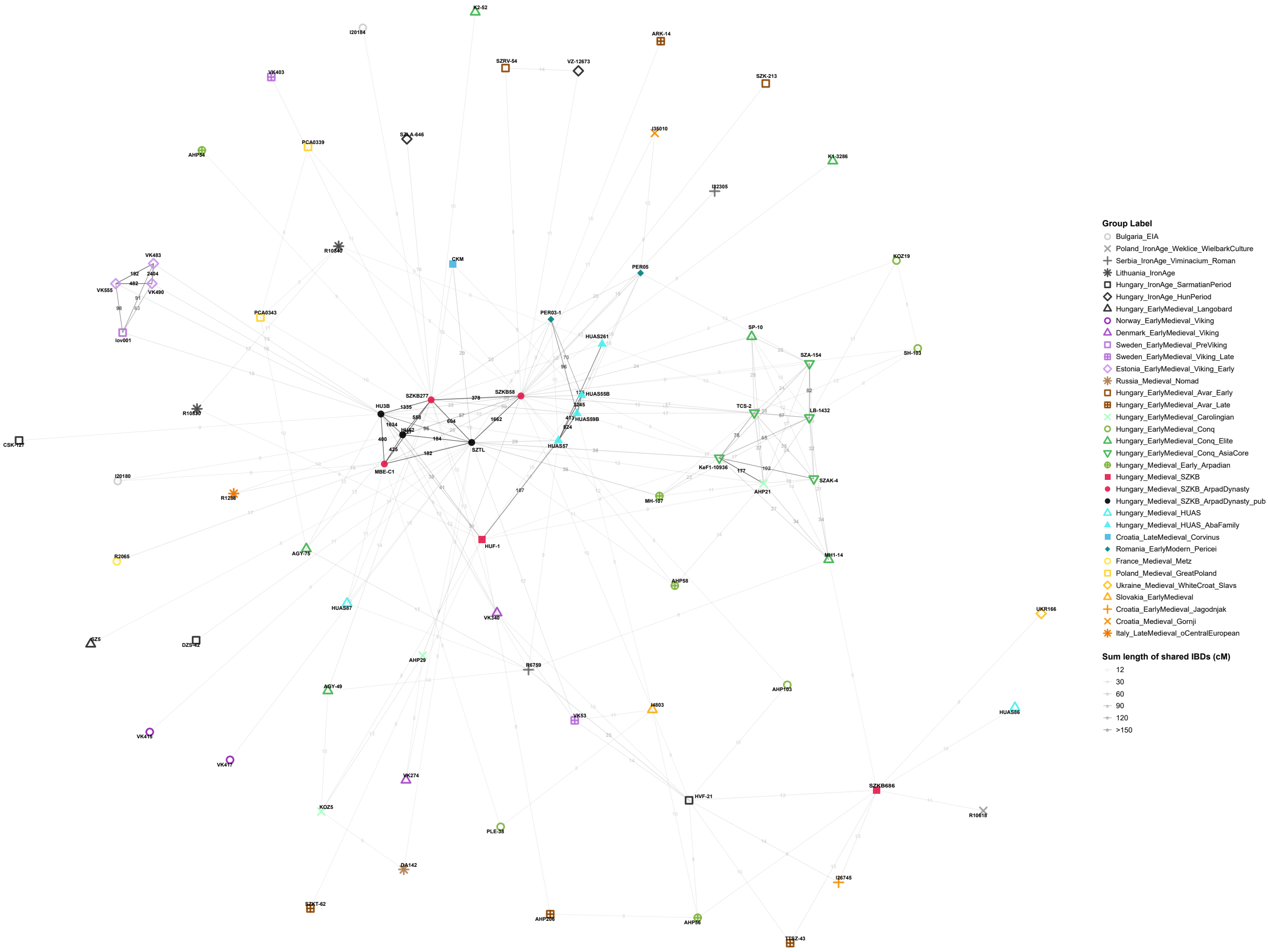
Group Label
Bulgaria_EIA
Poland_IronAge_Weklice_WielbarkCulture
Serbia_IronAge_Viminacium_Roman
Lithuania_IronAge
Hungary_IronAge_SarmatianPeriod
Hungary_IronAge_HunPeriod
Hungary_EarlyMedieval_Langobard
Norway_EarlyMedieval_Viking
Denmark_EarlyMedieval_Viking
Sweden_EarlyMedieval_PreViking
Sweden_EarlyMedieval_Viking_Late
Estonia_EarlyMedieval_Viking_Early
Russia_Medieval_Nomad
Hungary_EarlyMedieval_Avar_Early
Hungary_EarlyMedieval_Avar_Late
Hungary_EarlyMedieval_Carolingian
Hungary_EarlyMedieval_Conq
Hungary_EarlyMedieval_Conq_Elite
Hungary_EarlyMedieval_Conq_AsiaCore
Hungary_Medieval_Early_Arpadian
Hungary_Medieval_SZKB
Hungary_Medieval_SZKB_ArpadDynasty
Hungary_Medieval_SZKB_ArpadDynasty_pub
Hungary_Medieval_HUAS
Hungary_Medieval_HUAS_AbaFamily
Croatia_LateMedieval_Corvinus
Romania_EarlyModern_Pericei
France_Medieval_Metz
Poland_Medieval_GreatPoland
Ukraine_Medieval_WhiteCroat_Slavs
Slovakia_EarlyMedieval
Croatia_EarlyMedieval_Jagodnjak
Croatia_Medieval_Gornji
Italy_LateMedieval_oCentralEuropean
Sum length of shared IBDs (cM)
12
30
60
90
120
>150

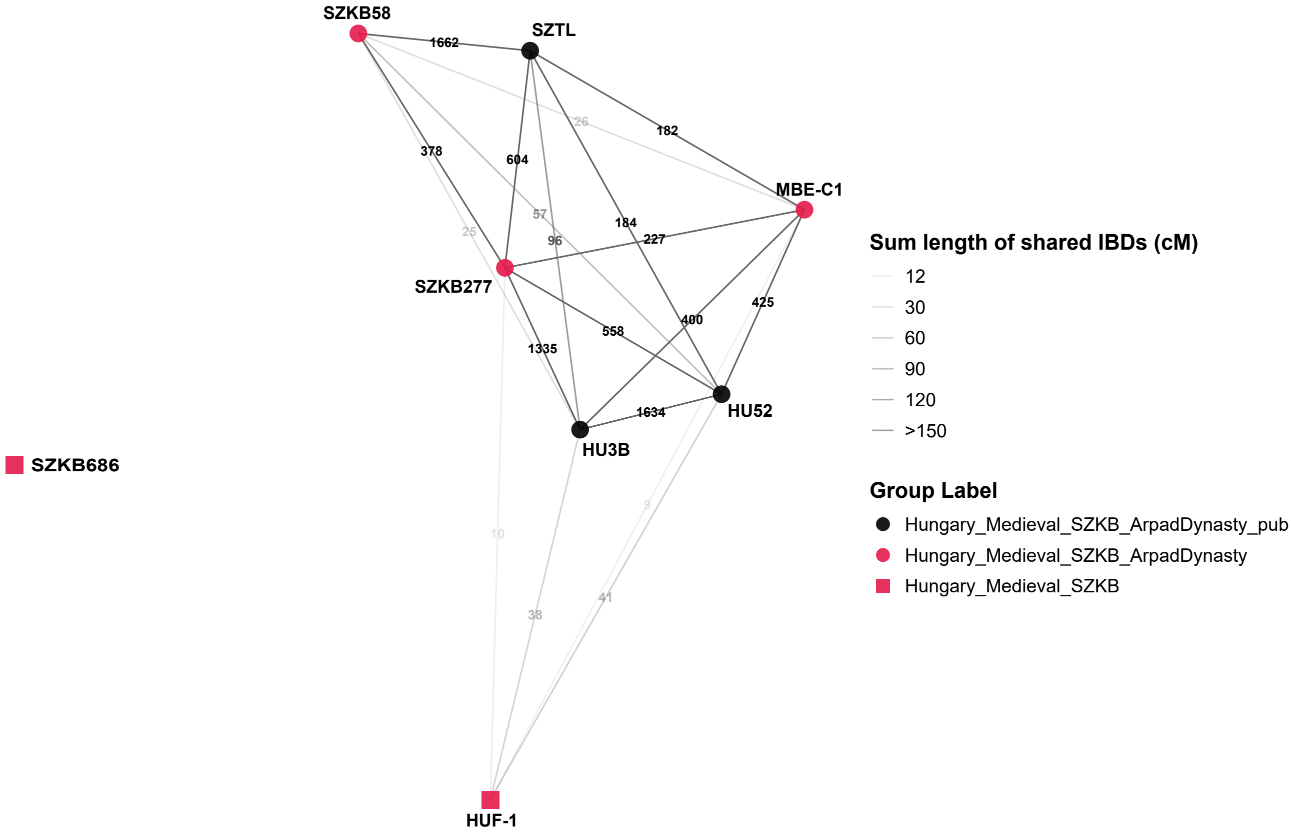
SZKB58
SZTL
1662
26
182
378
604
MBE-C1
57
184
25
96
227
SZKB277
425
400
558
1335
HU52
1634
HU3B
SZKB686
9
10
41
38
HUF-1
Sum length of shared IBDs (cM)
12
30
60
90
120
>150
Group Label
Hungary_Medieval_SZKB_ArpadDynasty_pub
Hungary_Medieval_SZKB_ArpadDynasty
Hungary_Medieval_SZKB

A

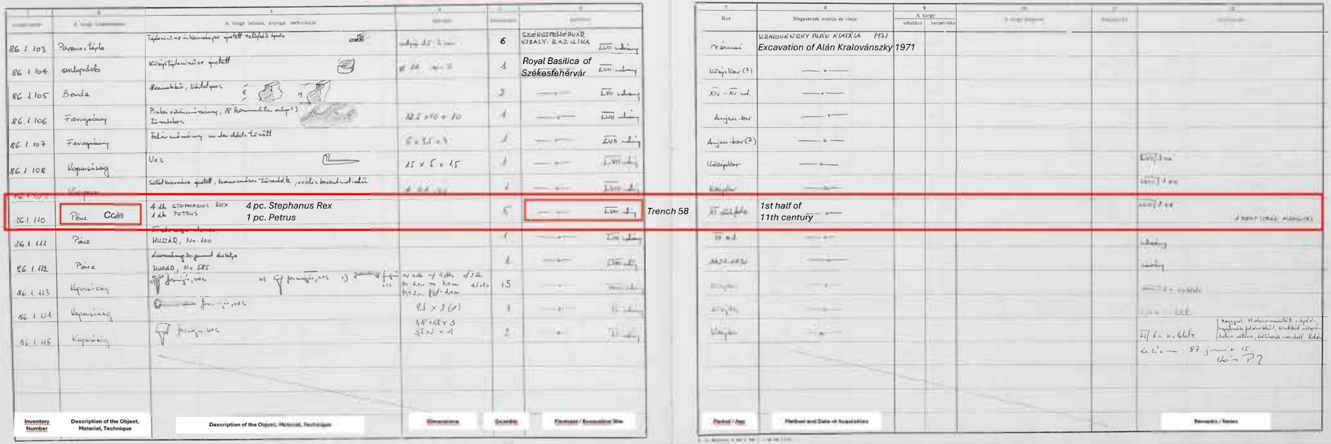
Archaeological Inventory Book - Régészeti
leltárkönyv
Excavation of Alán Kralovánszky 1971
Royal Basilica of Székesfehérvár
4 pc. Stephanus Rex
1 pc. Petrus
Trench 58
1st half of 11th century
Inventory Number
Description of the Object, Material, Technique

B

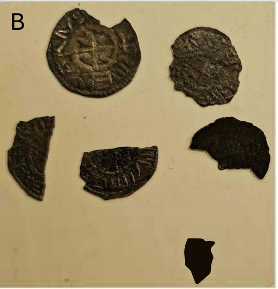

Supplementary Figure Legends

Figure S1. Burial site of Béla III and his wife at Székesfehérvár. Lithograph by Érdy (1853) showing the original tomb arrangement, with a schematic identifying the four occupant positions: HU52 (likely Andrew of Halych), HU3B (Béla III), HUAA (Anna of Antioch), and HUF1 (the fetus from the adjacent tomb).

Figure S2. Branching of the R-Z2123 Y-chromosomal haplogroup. Phylogenetic tree showing the position of the Árpád Dynasty (R-ARP) sub-clades within the broader R-Z2123 lineage, with TMRCA estimates (years before present) for each branch and assignment of identified individuals to specific R-ARP sub-haplogroups.

Figure S3. The paternal lineages of Dmitry Alexandrovich and Béla, Duke of Macsó. Genealogical chart showing both lines of descent from Yaroslav the Wise, demonstrating the Rurikid affiliation of MBE-C1 (Béla of Macsó).

Figure S4. Relatedness of the ancestors of Béla II and Béla III, and HU52. Genealogical reconstruction of the Árpád lineage showing the relevant ancestors of the analyzed individuals.

Figure S5. IBD network at the 8 cM threshold. The full IBD network of individuals sharing at least 8 cM of cumulative IBD segments with the R-ARP-positive individuals and the fetus, including more distantly connected individuals than shown in Figure 4 (12 cM threshold).

Figure S6. IBD intragroup connections. The IBD network restricted to connections among the nine Árpád-related individuals, with edge weights indicating sum length of shared IBD segments.

Figure S7. Coins found adjacent to SZKB58. (A) Excerpt from the Archaeological Inventory Book of Alán Kraloványszky (1971) describing the five coins found adjacent to the remains of SZKB58, identified as 4 coins of Stephanus Rex and 1 of Petrus, dated to the first half of the 11th century. (B) Photograph of the five coins.
